# Supplementary material for: In vitro to in vivo acetaminophen hepatotoxicity extrapolation using classical schemes, pharmacodynamic models and a multiscale spatial-temporal liver twin
Source: Front Bioeng Biotechnol. 2023 Feb 2;11:1049564. doi: 10.3389/fbioe.2023.1049564 (PMC9932319; doi:10.3389/fbioe.2023.1049564)
Supplement: Supplementary file 1 [file DataSheet2.PDF]

frontiers

# *Supplementary Material*

## 1 Supplementary to the material and methods

### 1.1 *In vitro*-PD cell metabolic model of APAP

The rationale for the set of reactions considered in this work was presented in the main text. The processes involved in APAP toxicity can be found in greater details in following standard references (Jaeschke et al., 2012; Klaassen et al., 2013) We assume that the enzyme-catalyzed reactions follow Michaelis-Menten kinetics. When several substrates are present, the reaction rate formula depends on the mechanism. For UGT (Hochman et al., 1981), SULT (Banerjee and Roy, 1968; Duffel and Jakoby, 1981; Chapman et al., 2004; Allali-Hassani et al., 2007; Tyapochkin et al., 2008) and GST (Armstrong, 1991; Phillips and Mantle, 1991), the precise mechanism is not known. For simplicity we assume the following general form:

$$v = V_{max} \frac{AB}{(K_A + A)(K_B + B)}$$

which is valid for several ordered and non-ordered bi-substrate types of reactions (Leskovac, 2004) Finally, to take into account the feedback of JNK on ROS production, the production of ROS triggered by NAPQI adducts is modeled by a Hill formula. The membrane permeability transition (MPT) is also modeled as a cooperative mechanism. The non-linear ordinary differential equations to model those reactions are given here after.

$$\frac{d[APAP]_{cell}}{dt} = \frac{P \cdot S}{V_{cell}}([APAP]_{outside} - [APAP]_{cell}) - v_{CYP2E1} - v_{CYP1A2} - v_{UGT} - v_{SULT}$$

$$\frac{d[NAPQI]}{dt} = -k_{on}[NAPQI] + v_{CYP2E1} + v_{CYP1A2} - v_{GST}$$

$$\frac{d[NAPQI_{bound}]}{dt} = k_{on}[NAPQI]$$

$$\frac{d[PAPS]}{dt} = k_{p,PAPS} - k_{d,PAPS}[PAPS] - v_{SULT}$$

$$\frac{d[UDPg]}{dt} = k_{p,UDPg} - k_{d,UDPg} - v_{UGT}$$

$$\frac{d[GSH]}{dt} = k_{p,GSH} - k_{d,GSH}[GSH] - v_{GST} - v_{ROSd}$$

$$\frac{d[ROS]}{dt} = v_{ROSp} - v_{ROSd}$$

$$\frac{d[Mito]}{dt} = k_{p,Mito} - k_{d,Mito}[Mito] - v_{MPT}$$

$$\frac{d[ATP]}{dt} = k_{p,ATP}[Mito] - k_{d,ATP}[ATP]$$

With the following expressions for the reactions rates:

$$v_{CYP2E1} = V_{max,CYP2E1} \frac{[APAP]_{cell}}{[APAP]_{cell} - K^m_{CYP2E1}}$$

$$v_{CYP1A2} = V_{max,CYP1A2} \frac{[APAP]_{cell}}{[APAP]_{cell} - K^m_{CYP1A2}}$$

$$v_{GST} = V_{max,GST} \frac{[GSH][NAPQI]}{\left(K^m_{GST,NAPQI} + [NAPQI]\right) \cdot \left(K^m_{GST,GSH} + [GSH]\right)}$$

$$v_{UGT} = V_{max,UGT} \frac{[UDPg][APAP]_{cell}}{\left(K^m_{UGT,APAP} + [APAP]_{cell}\right) \cdot \left(K^m_{UGT,UDPg} + [UDPg]\right)}$$

$$v_{SULT} = V_{max,SULT} \frac{[PAPS][APAP]_{cell}}{\left(K^m_{SULT,APAP} + [APAP]_{cell}\right) \cdot \left(K^m_{SULT,PAPS} + [PAPS]\right)}$$

$$v_{ROSp} = V_{max,ROSp} \frac{[NAPQI_{bound}]^{n1}}{[NAPQI_{bound}]^{n1} + \left(K^m_{ROSp}\right)^{n1}}$$

$$v_{ROSd} = V_{max,ROSd} \frac{[GSH][ROS]_{cell}}{\left(K^m_{ROSd,ROS} + [ROS]_{cell}\right) \cdot \left(K^m_{ROSd,GSH} + [GSH]\right)}$$

$$v_{MPT} = V_{max,MPT} \frac{[ROS]^{n2}}{[ROS]^{n2} + (K^m_{MPT})^{n2}}$$

$P$ is the permeability of the cells for APAP, $S$ the effective contact surface of the cells with the blood compartment, $V_{cell}$ is the hepatocyte volume. $[X]$ denotes the concentration of species X. $[APAP]_{outside}$ is chosen as a linear decreasing function in time to account for the low cell uptake of APAP in the *in vitro* culture. This allows to uncouple the external field with the intracellular reactions. The simulations are then run for 3215 cells (approximate number of cells in a representative liver lobule), and the fraction of dead cells at time of interests are computed.

### 1.2 *In vivo*-PD cell metabolic model of APAP

Compared to the *in vitro* cell, the *in vivo* cell metabolic model differs in the CYP2E1 and CYP1A2 activities, which were measured to be respectively 3.3 and 1.8 times higher *in vivo* than *in vitro*. The maximal reaction rates $V_{max,CYP2E1}$ and $V_{max,CYP1A2}$ were thus modified accordingly. Similarly, the initial concentration of GSH was measured to be 2 times lower *in vivo* than *in vitro* which was also changed accordingly in the *in vivo* model of the cell. I.e., $\frac{V^{vivo}_{max,CPY2E1}}{V^{vitro}_{max,CPY2E1}} = 3.2$, $\frac{V^{vivo}_{max,CPY1A2}}{V^{vitro}_{max,CPY1A2}} = 1.8$, $\frac{[GSH]^{vivo}(t=0)}{[GSH]^{vitro}(t=0)} = 2$.

The external profile of APAP, $[APAP]_{outside}$ is then chosen according to the different strategies described in the main text.

### 1.3 *In vivo* pharmacokinetic model

A simple pharmacokinetic model (PK) was used to model the kinetics of the concentration of APAP after injection in the peritoneum. The model considers first-order absorption and a linear elimination from the blood systemic circulation. A dose dependent bioavailability effect where only a fraction of the initial dose in the peritoneum reaches the systemic blood concentration was also assumed. There are thus two compartments under study: the peritoneum and the blood compartments. The equations for both compartments are given as follow:

$$\frac{dn_p}{dt} = -k_p n_p = -F k_p n_p - (1-F) k_p n_p$$
$$\frac{dn_b}{dt} = F k_p n_p - k_b n_b$$

With $n_p$ and $n_b$ the number of molecules in the peritoneum and the systemic blood compartments, respectively, $k_p$ the rate of removal from the peritoneum compartment, $F = \frac{n_0^\gamma}{n_0^\gamma + D_{50}^\gamma}$ the bioavailability fraction (determining the effective fraction of APAP molecules that reach the blood compartment). In the latter, $n_0 = n_p(t=0)$ is the initial dose (in µmol), $\gamma$ the Hill coefficient, and $D_{50}$the dose for which bioavailability is half of the maximal bioavailability. The solution of this system of equations reads as

$$n_b(t) = F n_0 \frac{k_p}{k_b - k_p}\left(e^{-k_p t} - e^{-k_b t}\right)$$

As we are interested in the concentration of APAP in blood, we need to divide the number of APAP molecules in the blood compartment by a volume. The corresponding parameter is the volume of distribution (i.e. total volume of blood, $V_b$), leading to the following expression of the APAP concentration

$$[APAP]_b(t) = \frac{F n_0}{V_b} \frac{k_p}{k_b - k_p}\left(e^{-k_p t} - e^{-k_b t}\right)$$

This model has 5 parameters to calibrate: $k_p, k_b, \gamma, D_{50}, V_b$.

### 1.4 *In vivo*-PD compartment model of APAP

To adapt to the *in vivo* case, the profile of APAP concentration outside of the cells can no longer be considered as an independent input function but is replaced by a coupled Pharmacokinetic/Pharmacodynamic model. The model considers the initial injection in the peritoneum, release in the blood vasculature, elimination by other organs (e.g. the kidneys), modeled as taking place homogeneously in the systemic blood compartment, as well as uptake by the cells and elimination by the intracellular reactions, the latter following the same ODEs of the in-vitro model. As in the in-vitro case, the simulations were run for 3215 cells and the total uptake was corrected for the total number of cells in the liver. The equations read as follow:

$$\frac{dn_p}{dt} = -k_p n_p$$

$$\frac{dn_b}{dt} = F k_p n_p + \frac{N_{liver}}{N_{lob}} PS \sum_{j}^{N_{lob}} \left( [APAP]_{cell}^{j} - \frac{n_b}{V_b} \right) - k_{others}\, n_b$$

$$\frac{d[APAP]_{cell}^{j}}{dt} = -\frac{PS}{V_{cell}} \left( [APAP]_{cell}^{j} - \frac{n_b}{V_b} \right) - \sum_{i} v_i\left([APAP]_{cell}^{j}\right)$$

With $n_p$ and $n_b$ the number of molecules in the peritoneum and the systemic blood compartments, $k_p$ the first order rate of removal from the peritoneum compartment, $k_{others}$ the first order rate of removal from the blood compartment from effects other than the liver, $F$ the bioavailability fraction (determining the effective fraction of APAP molecules that reach the blood compartment), $N_{liver}$ the number of cells in the liver, $N_{lob}$ the number of cells in the considered representative (piece of a) liver lobule, $V_b$ the total volume of blood, $k_{others}$ the rates of removal from other organs (e.g. kidneys), $V_{cell}$ the volume of a hepatocyte, $j$ the index enumerating the cells in the representative lobule piece. Finally, $v_i$ are reactions identical to the in-vitro case with $v_1 = v_{CYP2E1}, v_2 = v_{CYP1A2}, v_3 = v_{UGT}, v_4 = v_{SULT}$.

The rest of the ODEs are identical to the in-vitro case. The parameters values are the same as in the *in vitro* case except for the $V_{max,CPY2E1}$ and $V_{max,CPY1A2}$ and the GSH initial concentration as described previously. $N_{lob} = 3215$ is chosen according to the number of hepatocytes (liver parenchyma cells) in a representative liver lobule piece (Hoehme et al., 2010). Considering C57BL/6N mice with liver weight of 1.95g (MPD: Data set: Reed2, 2), $1.35 \cdot 10^8$ hepatocytes per gram liver (Sohlenius-Sternbeck, 2006) one can estimate the number of hepatocytes in the liver as: $N_{liver} = 1.95 \cdot 1.35 \cdot 10^8 = 2.6325 \cdot 10^8$ hepatocytes, which, divided by the number of hepatocytes per simulated lobule piece, results in $N_{liver}/N_{lobule} \approx 80870$ . Otherwise, from dividing the liver volume of ~*1.3 mL* as reported in ref. (Xie et al., 2014) by the volume of the lobule piece, $V_{lobule} \approx 52.5 \times 10^{-3} mm^3 = 52.5 \times 10^{-6} mL$ (Hoehme et. al., 2010), hence the volume fraction of a simulated lobule piece is. $V_{liver}/V_{lobule} \approx 25000$. Approximating the liver mass density by that of water, the liver volume value of ~1.35 mL indicates a liver weight of ~1.35g but even correcting for the difference to the previously mentioned data set Reed2 by a factor of 1.95/1.35, one would obtain ~36000 lobule pieces instead of ~81000 for the ratio of liver volume and liver lobule piece volume. As a cross-verification, with a hepatocyte volume of $V_{hepat} \approx 1.26 \times 10^{-5} mm^3$, and a volume fraction of hepatocyte of ~*80-81%* (Hammad et. al, 2014), the simulated liver lobule piece volume corresponds to about ~3350 hepatocytes i.e., about 4% above our value of 3215 cells. Assuming $N_{liver}/N_{lobule} \approx V_{liver}/V_{lobule} \approx 25000$, indicates that the range for $N_{liver}/N_{lobule} \approx 25000 - 80870$ . We conclude that there is quite some uncertainty is in the size of the mouse that correlates with its liver size, with the volume taken by the vessels at supra-lobular scale, the volume by the bile ducts etc., which may account for the variation.

## 1.5 *In vivo*-PD compartment model of APAP with storage in space of Disse

We also tested the influence of introducing a storage mechanism of APAP in the blood which would represent APAP storage in the ECM (extracellular matrix) and then release into the space of Disse and would thus impact the profile of the APAP blood concentration over time. The space of Disse

was not represented as a compartment per se and the storage mechanism was directly considered to take place in the blood compartment to keep the number of new parameters as low as possible. The full system of equations reads as

$$\frac{dn_p}{dt} = -k_p n_p$$

$$\frac{dn_b}{dt} = F\, k_p n_p - k_{others}\, n_b \ - k_{ECM}^{+} n_b \tilde{n}_{ECM} \ + k_{ECM}^{-} n_{b,ECM} + PS\frac{N_{liver}}{N_{lob}}\sum_{j}^{N_{lob}}\left([APAP]_{cell}^{j} - \frac{n_b}{V_b}\right)$$

$$\frac{d\tilde{n}_{ECM}}{dt} = -k_{ECM}^{+} n_b\, \tilde{n}_{ECM} + k_{ECM}^{-} n_{b,ECM}$$

$$\frac{dn_{b,ECM}}{dt} = k_{ECM}^{+} n_b \tilde{n}_{ECM} \ - k_{ECM}^{-} n_{b,ECM}$$

$$\frac{d[APAP]_{cell}^{j}}{dt} = -\frac{PS}{V_{cell}}\left([APAP]_{cell}^{j} - \frac{n_b}{V_b}\right) - \sum_{i} v_i\left([APAP]_{cell}^{j}\right)$$

$$F = \frac{n_0^{\gamma}}{n_0^{\gamma} + D_{50}^{\gamma}}$$

$\tilde{n}_{ECM}$ denotes the number of unoccupied binding sites, $n_{b,ECM}$ the number of occupied ECM sites measured in moles APAP bound to ECM, whereby an APAP molecule bounded to an ECM-site may be interpreted as APAP-ECM-site-complex. Consequently, $k_{ECM}^{+}$ is the rate of binding to free ECM sites, and $k_{ECM}^{-}$ the rate of unbinding from ECM sites (dissociation of the complex).

This set of equations can be simplified if we assume that the number of binding sites is large enough compared to the number of sites where APAP effectively binds to (infinite reservoir hypothesis), so that it can be considered constant over time.

If we define a new quantity $\tilde{k}_{ECM}^{+} = k_{ECM}^{+} \tilde{n}_{ECM}(t = 0)$ the effective rate of binding to free ECM sites, the equation over $\tilde{n}_{ECM}(t = 0)$ can be dropped and the remaining equations simplify

$$\frac{dn_p}{dt} = -k_p n_p$$

$$\frac{dn_b}{dt} = F\, k_p n_p - k_{others}\, n_b \ - \tilde{k}_{ECM}^{+} n_b + k_{ECM}^{-} n_{b,ECM} + PS\frac{N_{liver}}{N_{lob}}\sum_{j}^{N_{lob}}\left([APAP]_{cell}^{j} - \frac{n_b}{V_b}\right)$$

$$\frac{dn_{b,ECM}}{dt} = \tilde{k}_{ECM}^{+} n_b - k_{ECM}^{-} n_{b,ECM}$$

$$\frac{d[APAP]_{cell}^{j}}{dt} = -\frac{PS}{V_{cell}}\left([APAP]_{cell}^{j} - \frac{n_b}{V_b}\right) - \sum_{i} v_i\left([APAP]_{cell}^{j}\right)$$

$$F = \frac{n_0^{\gamma}}{n_0^{\gamma} + D_{50}^{\gamma}}$$

At $t = 0, n_p(0) = n_0,\ n_b(0) = 0,\ n_{b,ECM}(0) = 0, [APAP]^j{}_{cell}(0) = 0.$

This model introduces two additional parameters: $\tilde{k}_{ECM}^{+}$ and $k_{ECM}^{-}$.

### 1.6 Spatio-temporal *in vivo* model of APAP

As mentioned in main text, the spatio-temporal consists in three aspects *(i)* stationary blood flow modeling on the liver lobule, *(ii)* modeling of transport of molecules in the vasculature of the liver lobule, *(iii)* intracellular ODEs inside each cell of the liver lobule and *(iv)* coupling of the liver lobule to the systemic circulation. As *(iii)* is identical to the previous section, only *(i, ii, iv)* will thus be presented in the following sections.

#### 1.6.1 Blood flow modeling equations

Along a sinusoidal segment with its two ends denoted as indices $i$ and $j$ , Poiseuille flow is considered.

Typically, a segment links branching points $i$ and $j$. The Poiseuille flow then reads:

$$R_{i,j}Q_{i,j} = \Delta P_{i,j} = P_i - P_j,$$

where $R_{i,j} = \frac{8\eta_{i,j}(D)L_{i,j}}{\pi\, r_{i,j}^4}$ is the hydraulic resistance with $L_{i,j}$ the length of the sinusoid segment, $r_{i,j}$ the radius of the sinusoid and $\eta_{i,j}$ is the apparent blood viscosity with a diameter dependence to account for the Fåhræus-Lindqvist effect. We chose the well-established model by Secomb and Pries (Secomb and Pries, 2013), with the following expression of the blood viscosity:

$$n_{i,j}(D) = \left[1 + (\eta_{45} - 1)\frac{(1 - H_D)^C - 1}{(1 - 0.45)^C - 1}\left(\frac{D}{D - 1.1}\right)^2\right]\left(\frac{D}{D - 1.1}\right)^2$$

$$\eta_{45} = 6\exp(-0.085\, D) + 3.2 - 2.44\exp\left(-0.06\, D^{0.0645}\right)$$

$$C = (0.8 + \exp(-0.075\, D))(-1 + (1 + 10^{-11}D^{12})^{-1}) + (1 + 10^{-11}D^{12})^{-1}$$

With $\eta_{45}$ the relative apparent viscosity for a fixed discharge hematocrit $H_D$. This is a simplification as one should consider in addition a phase separation effect (Secomb, 2017) but as its influence on flow can be neglected (Guibert et al., 2010; Boissier et al., 2021), a constant discharge hematocrit $H_D = 0.45$ was considered.$Q_{i,j}$ is the blood flow on the sinusoid and $\Delta P_{i,j} = P_i - P_j$ the pressure difference at both nodes of the vascular graph.

For all non-boundary vascular nodes $i$, the mass conservation relation implies that the sum of all signed fluxes of connected vessels at node $i$ is zero, namely:

$$\sum_{j \in neighbors(i)} Q_{i,j} = 0.$$

We set flow boundary conditions at the entrance of the virtual liver lobule, i.e. $Q_{lob,in} = 1.2 \cdot 10^6 \mu m^3/s$, and a 0 pressure boundary condition in the CV such that sinusoid velocities and PV-CV pressure gradient are close to reported values. More explicitly, the input lobular flow is such that each portal vein vascular node $Q_{PV,i}$ has the same inflow.

$$Q_{PV,i} = \frac{Q_{lob,in}}{\#PV\ nodes}$$

Where $\#PV\ nodes$ is the total number of portal vein nodes of the lobule.

This follows the procedure described in (Boissier et al., 2021) which gathered data from literature (see Table 4 in that reference). The average sinusoid velocity value obtained is 48 µm/s which is close to the data for mice with intravital imaging (60-75 µm/s). The PV-CV gradient obtained is 116.5 Pa again in line with the available data (26.6-540 Pa) for rats.

Different from (Boissier et al., 2021) we here assume that a lobule obtains inflow from three portal veins (and hepatic arteries) located in three of its corners, as indicated in the below scheme. As a portal vein / hepatic artery unit feeds simultaneously three adjacent liver lobule pieces, the number of feeding portal vein / hepatic artery units feeding a lobule is 3 x 1/3 = 1.

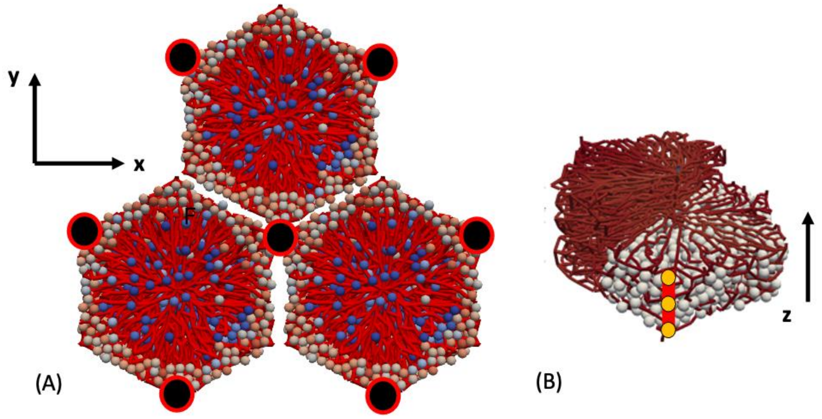


**Scheme:** (A) arrangement of lobules with one portal and hepatic vein (back dot with red circle) feeding its blood into three adjacent lobule pieces. (B) Unit composed of one portal and hepatic vein (red vertical line) with three nodes (yellow dots).

### 1.6.2 Transport modeling equations in a liver lobule piece

Advection-reaction transport equations were considered where diffusion effect inside the vasculature neglected. Cells on the border of the hepatocytes were considered as source terms in a multi-phase approach, where the uptake is done by passive diffusion. This leads to the following set of equations:

$$\frac{\partial C_{blood}(x)}{\partial t} + \nabla(\vec{v}\, C_{blood}(x)) = -\sum_{i \in neighbournig\_cells(x)} PS(C_{blood}(x) - C_{cell}^{i}),$$

where $C_{blood}$ and $C_{cell}$ are the concentration of metabolites transported in blood (here, only APAP) and the concentration in the cell $i$ in contact with the sinusoid at point $x$, $\vec{v}$ is the stationary velocity field, computed in the previous section. $neighbournig\_cells(x)$ is the set of all cells in direct contact with the sinusoid at point $x$. Since this is a hyperbolic partial differential equation, one only

needs to prescribe inlet boundary condition, i.e. at the portal veins of the liver lobule, which will be described in the next section. $PS$ is again the permeability-surface product (see above).

The exchange surface of the hepatocytes is the same for all hepatocytes in representative liver lobule. Hepatocytes are considered as cubes, micro-vili are assumed to increase the effective exchange surface by a factor 6 and the fraction of the average surface of the hepatocyte in contact with sinusoids is 22% (Hammad et al., 2014). Its definition thus reads as: $S = 6 \cdot (2\ R_h^2) \cdot 0.22 \cdot 6 = 4300\ \mu m^2$. The exchange surface of a single hepatocyte with its connected vascular nodes is then distributed equally among each vascular node as:

$$S_n = \frac{S}{\#connected\_nodes}$$

Where $\#connected\_nodes$ is the number of connected nodes and $S_n$ is the exchange surface of the hepatocyte with one connected nodes.

A geometric criterion is computed to account if a cell is connected to a vascular node or not:

$$||X_h - X_n|| \leq \sqrt{2} \cdot (R_h + R_n)$$

Here, $X_h$ is the position of the hepatocyte, $X_n$ the position of a vascular node, $R_h$ the radius of the hepatocyte and $R_n$ the (activity) radius of the node (i.e. radius of the cylinder to which it belongs as the sinusoids all have the same radius).

Note that there is the space of Disse between the sinusoids and the sinusoids aligning them. As the sinusoidal diameter, the size of the Disse space (~ 0.5um) may vary such that a precise interface area at these small scales cannot and has not been determined.

### 1.6.3 Coupling of liver lobule to other compartments

Let us first consider the liver as a compartment and the kidneys (and other organs) effect as homogeneous within the systemic blood compartment (no spatial effect). Assuming transport from one compartment to the other by flow only, this leads to the following set of equation:

$$\frac{dn_p}{dt} = -k_p n_p$$

$$\frac{dn_{blood}}{dt} = F k_p n_p + Q_{liver}\frac{n_{liver}}{V_{liver}} - k_{others} n_{blood} - Q_{liver}\frac{n_{blood}}{V_{blood}}$$

$$\frac{dn_{liver}}{dt} = Q_{liver}\left(\frac{n_{blood}}{V_{blood}} - \frac{n_{liver}}{V_{liver}}\right) - \sum_i v_i \left(\frac{n_{liver}}{V_{liver}}\right)$$

Here, $Y_x$ with $Y \in \{Q, n, V\}$ (= volume flow rate, number of APAP molecules and the compartment volume, respectively) denote the amount of quantity Y in compartment $x \in \{liver, blood, others, p\}$. Now, let's replace the liver compartment by spatio-temporal liver lobules

as described by the previous sections. The schematic representation below illustrates the coupling of the liver lobule model to compartments, the liver lobules being organized in parallel to each other. Again, the effect of kidneys is assumed to be homogeneous within the systemic blood compartment (represented as the connection between peritoneum and the liver).

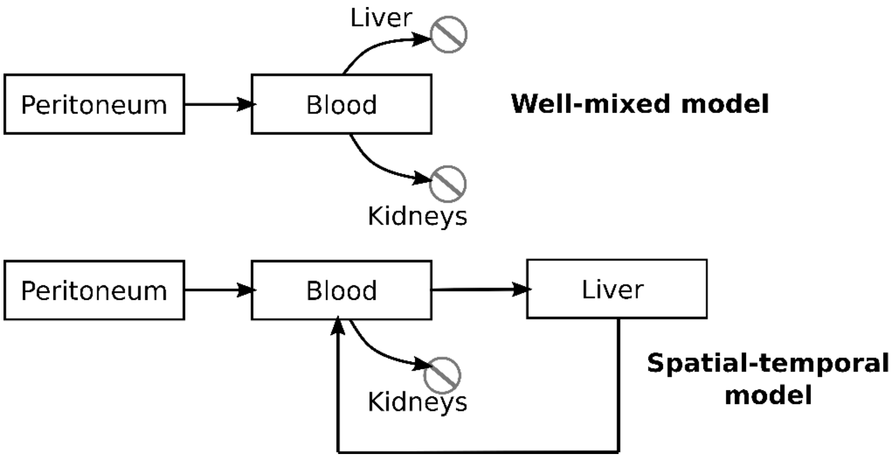


**Scheme:** Comparison of well-mixed approach where APAP is transfer directly from the blood compartment to the hepatocytes (first row) and spatial-temporal approach where liver is treated as an additional compartment.

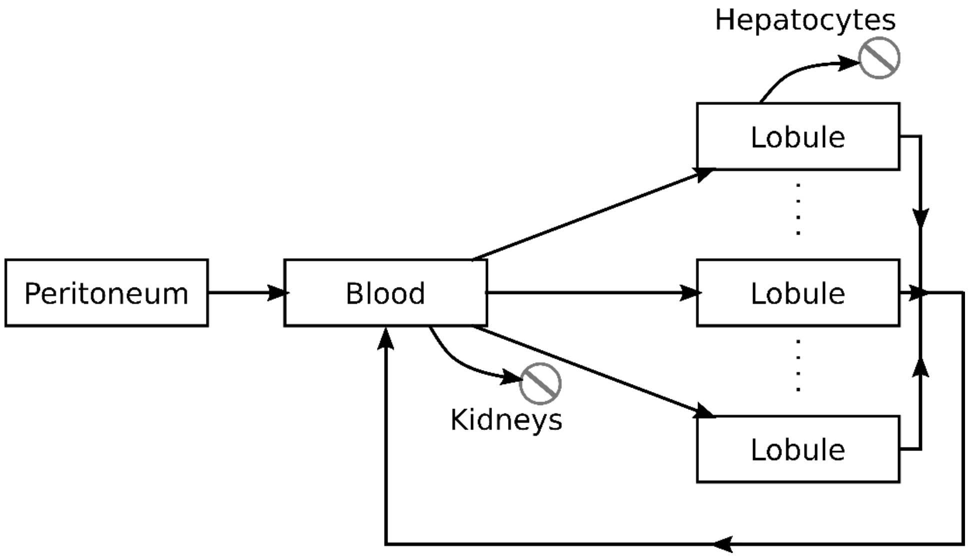


**Scheme:** spatial-temporal model: the liver is modeled as parallel arrangement of lobules.

Equality of rates of mass transport are considered to relate the central vein (liver outlet) concentration to the liver concentration:

$$\sum_{i}^{N_{lobule}} Q_{lob,CV}^{i} \frac{n_{lob}^{i}(CV)}{V_{CV}^{i}} = Q_{liver} \frac{n_{liver}}{V_{liver}}$$

With $N_{lobule}$ the number of liver lobules, $Q_{lob,CV}^{i}$ the flow rate of lobule $i$ leaving through the central vein, $n_{lob}^{i}(CV)$ the amount of moles at the outlet of the central vein of lobule $i$, $V_{CV}^{i}$ the volume of the

central vein of liver lobule $i$, $Q_{liver}$ the liver flow rate, $n_{liver}$ the number of moles at the inlet of the liver, $V_{liver}$ the volume of the liver.

And similarly for the portal vein (liver inlets):

$$\sum_{i}^{N_{lobule}} \sum_{j}^{N_{PV}} Q^{i}_{lob,PV_j} \frac{n^{i}_{lob}\,(PV_j)}{V^{i}_{PV_j}} = Q_{liver} \frac{n_{blood}}{V_{blood}}$$

With $Q^{i}_{lob,PV_j}$the flow rate entering the portal vein $j$ of liver lobule $i$, $n^{i}_{lob}(PV_j)$ the amount of moles at the inlet of the portal vein $j$ of lobule $i$, $V^{i}_{PV_j}$ the volume of the portal vein $j$ of liver lobule $i$, $n_{blood}$ the number of moles in the blood compartment, $V_{blood}$ the volume of the blood compartment.

Assuming that all liver lobules are similar, one can only consider one lobule and set:

$$Q_{lob,CV} = \frac{Q_{liver}}{N_{lobule}}$$

$$\frac{n_{lob}(CV)}{V_{CV}} = \frac{n_{liver}}{V_{liver}}$$

At the outlet of a single liver lobule, and similarly for inlet outlet:

$$\sum_{j}^{N_{PV}} Q_{lob,PV_j} = \frac{Q_{liver}}{N_{lobule}}$$

$$\frac{n_{lob(PV_j)}}{V_{PV_j}} = \frac{n_{blood}}{V_{blood}}$$

The flow is then distributed equally among all portal veins. For the spatio-temporal liver lobule, we thus have boundary conditions to set and the equations of the other compartments coupled to the spatio-temporal model thus become:

$$\frac{dn_p}{dt} = -k_p n_p$$

$$\frac{dn_{blood}}{dt} = Fk_p n_p + Q_{liver}\left(\frac{n_{lob}(CV)}{V_{CV}} - \frac{n_{blood}}{V_{blood}}\right) - k_{others} n_{blood}$$

This model thus introduces an additional parameter: the blood flow entering/leaving the liver, $Q_{liver}$ . Remember that $Q_{liver} = N_{lobule} Q_{lob,CV}$ i.e., the number of lobule pieces multiplied by the volume flow rate leaving the liver lobule piece via its central vein.

### 1.6.4 Estimation of liver flow rate

We set flow boundary conditions at the entrance of the virtual liver lobule $Q_{lob,in}$ in order to have sinusoid velocities close to what was measured experimentally in the literature as in reference (Boissier et al., 2021)(see end of SI section1.6.1). If one makes the simplifying assumption that all liver lobules are distributed in parallel (as in the previous subsection), one has the following expression for the liver flow rate:

$$Q_{liver} = \sum_{i=1}^{N_{lobule}} Q_{lob,in}^{i}$$

Here $N_{lobule}$ is the total number of virtual lobules in the liver and $Q_{lob,in}^{i}$ the flow rate at the entrance of the liver lobule $i$ distributed equally among all portal veins' nodes. Because of mass conservation, this is equal to the flow at the outlet of the liver lobule, i.e. equal to the sum of all flow at central veins' nodes. Assuming that all lobules have the same input flow rate and that a single lobule can be representative of the entire liver leads to:

$$Q_{liver} = N_{lobule} \cdot Q_{lob,in}$$

where $Q_{lob,in}$ is the input flow rate on the single representative lobule. With the boundary conditions on flow and pressure, and the geometry parameters determined from(Hoehme et al., 2010), this equation gives us a value $Q_{liver} = 5.34 mL/min$. Setting this value in the spatial-temporal model simulations results in an underestimation of APAP concentration in the blood compartment (Fig.7P-O). The simplest solution found to calibrate the spatial-temporal without changing either the architecture parameters (geometry and topology of the lobule piece) or the intracellular parameters was to change the number of lobules considered in the simulation, and see whether a simultaneous agreement of the simulated *in vitro* and pharmacokinetic data and their experimental counterparts could be obtained. Setting $Q_{liver} = 1.8\ mL/min$ captures the blood concentration data but overestimates the fraction of dead cells (Fig. 7R-S). Setting $Q_{liver} = 3.57 mL/min$ leads to a slight but acceptable underestimation of the APAP blood concentration data and a good agreement to the toxicity data (Fig. 7T-U). Finally, setting $Q_{liver} = 2.7 mL/min$ leads to a good agreement to all *in vivo* data sets (Fig. 7B-C). Note that all those volume flow rates are in line with experimental data reported in references (Davies and Morris, 1993; Schliess et al., 2014; Ghallab et al., 2016), which are 1.8 mL/min, 12 mL/min and 4mL/min, respectively.

### 1.6.5 Transition from well-mixed model with liver resolved blood compartment to well-mixed model with one total blood compartment

Keeping all parameters of the CL-3 model, and fixing the geometry and topology parameters of the liver lobule piece in the ST-model to those reported in (Hoehme et. al., 2010) and the boundary conditions for flow and pressure as in (Boissier et al., 2021), it was not possible to obtain a good simultaneous fit to the *in vitro* data and the pharmacokinetic data. Recall that compartmentalization in the ST-model occurs at the level of each individual hepatocyte i.e., each hepatocyte represents a spatially distinct chemical reaction volume arranged in a liver lobule micro-architecture, that is characterized by geometry and topology parameters. The coupling of the liver compartment to the extrahepatic blood compartment requires introduction of the liver volume flow rate $Q_{liver}$. In

contrast, in the CL-model 3, the reaction occurred in a single well-mixed compartment containing the entire systemic blood and all hepatocytes. No liver volume flow rate is necessary. In order to better understand the possible interplay of spatial compartmentalization and liver flow rate, we here study the simplest intermediate between the CL-model 3 and ST-model that would contain the liver volume flow rate $Q_{liver}$ as an explicit parameter. For this purpose, the mixed blood/liver compartment considered in CL-model 3 was split into one compartment representing the liver and one compartment representing the extrahepatic blood compartment. Despite this is certainly an oversimplification of the ST-model, it permits us some insight about the possible influence of the parameter "liver flow rate" that was present in the ST-model but not in CL-model 3.

We asked the question, whether we can identify a condition under which such a model, referred to CL-model 4 (a liver resolved model) could be approximated by a model of type CL-model 3, where only one compartment integrating liver and the entire systemic blood has been considered. The so resulting liver-resolved CL-model 4 would read as

$$\frac{dC_B}{dt} = \frac{Q_{liver}}{V_B}(C_{BL} - C_B)$$
$$\frac{dC_{BL}}{dt} = -\frac{Q_{liver}}{V_{BL}}(C_{BL} - C_B) - \frac{PS}{V_{BL}}(C_{BL} - C_H)$$

Here $C_B, C_{BL}$ and $C_H$ are the concentrations respectively of the external liver blood compartment, the liver blood compartment and the hepatocytes' compartment, respectively, where the CL-model 4 considers for simplicity the hepatocytes as represented by a single compartment, integrating all hepatocytes in one compartment, $V_B$and $V_{BL}$ are the volumes of the external liver blood and the liver blood, $Q_{liver}$ is the liver volume flow rate and $PS$ the permeability-surface product. We on purpose missed out the peritoneum and the APAP-decay term as well as the specific reactions controlling the APAP concentration in the hepatocyte compartment, as the core mechanism of the interplay between $Q_{liver}$ and the spatial compartmentalization does not depend on these specific terms and may (and below will) be studied even in the simplifying case of $C_H=0$.

We eliminate one volume variable and introduce rate variables, that later serve to define separate time scales, by setting $V^{\star} = \frac{V_{BL}}{V_B}$, $K_Q = \frac{Q_{liver}}{V_B}$ and $K_{PS} = \frac{PS}{V_B}$ . $K_Q$ and $K_{PS}$ determine the rates at which the molecules leave the blood compartment respectively by advection and diffusion. The equations the read:

$$\frac{dC_B}{dt} = K_Q(C_{BL} - C_B)$$
$$V^{\star}\frac{dC_{BL}}{dt} = -K_Q(C_{BL} - C_B) - K_{PS}(C_{BL} - C_H).$$

For simplicity we consider the initial condition that the external liver blood compartment was initially immediately filled with APAP and the other compartments are empty: $C_B(t=0) = C_0, C_{BL}(t=0) = 0, C_H(t=0) = 0$.

Note that there are two time scales associated with the volume flow rate and the permeability-surface product rate: $t_Q = \frac{1}{K_Q}$ and $t_{PS} = \frac{1}{K_{PS}}$. Consider now the case where the liver flow rate $Q_{liver} \gg PS \Leftrightarrow$

$K_{PS} \ll K_Q$ is much larger than the permeability surface product, the first characterizing the transported blood volume per unit of time along the sinusoid, the second the flux from blood into hepatocytes, hence $\frac{K_{PS}}{K_Q} = \epsilon \ll 1$ . This is equivalent to $t_Q \ll t_{PS}$. A rigorous time scale separation can be performed using the technique of singular perturbation analysis studying the long-and short time scale and later matching the solutions on both time scales within a matched asymptotic expansion. Let us first look at the short time scale, $t_Q$.

We non-dimensionalize the equations by introducing $C_B^\star = \frac{C_B}{C_0}, C_{BL}^\star = \frac{C_{BL}}{C_0}, C_H^\star = \frac{C_H}{C_0}$ and $t^\star = \frac{t}{t_Q}$.

This leads to

$$\frac{dC_B^\star}{dt^\star} = (C_{BL}^\star - C_B^\star)$$

$$V^\star \frac{dC_{BL}}{dt^\star} = -(C_{BL}^\star - C_B^\star) - \epsilon\,(C_{BL}^\star - C_H^\star)$$

Now if we assume asymptotic expansion for each concentration field: $C_i^\star = C_i^1 + \epsilon\, C_i^1 + O(\epsilon^2)$ and first gather the terms of leading order, this leads to

$$\boldsymbol{\frac{dC_B^0}{dt^\star} = C_{BL}^0 - C_B^0}$$

$$\boldsymbol{V^\star \frac{dC_{BL}^0}{dt^\star} = -C_{BL}^0 + C_B^0}$$

Note that we drop the "⋆" for convenience. This system of equations can be written in matrix form as:

$$\frac{d\boldsymbol{C}^0}{dt^\star} = A\boldsymbol{C}^0$$

Where $\boldsymbol{C}^0 = \begin{pmatrix} C_B^0 \\ C_{BL}^0 \end{pmatrix}$ and $A = \begin{bmatrix} -1 & 1 \\ \frac{1}{V^\star} & -\frac{1}{V^\star} \end{bmatrix}$. The eigenvalues and eigenvectors are given by

$$\lambda_1 = 0, \lambda_2 = -\left(\frac{1+V^\star}{V^\star}\right)$$

$$\boldsymbol{v}_1 = \begin{pmatrix} 1 \\ 1 \end{pmatrix}, \boldsymbol{v}_2 = \begin{pmatrix} -V^\star \\ 1 \end{pmatrix}$$

The solution then reads as

$$\boldsymbol{C}^0(t^\star) = \alpha_1 \boldsymbol{v}_1 + \alpha_2 \boldsymbol{v}_2 e^{-\left(\frac{1+V^\star}{V^\star}\right)t^\star}$$

Which means

$$C_B^0(t^\star) = \alpha_1 - \alpha_2 V^\star e^{-\left(\frac{1+V^\star}{V^\star}\right)t^\star},$$

$$C_{BL}^0(t^\star) = \alpha_1 + \alpha_2 e^{-\left(\frac{1+V^\star}{V^\star}\right)t^\star}.$$

The boundary conditions: $C_B^0(t^\star = 0) = 1, C_{BL}^0(t^\star = 0) = 0$ then lead to:

$$\alpha_1 = \frac{1}{1+V^\star}, \alpha_2 = -\left(\frac{1}{1+V^\star}\right).$$

And finally

$$C_B^0(t^\star) = \frac{1}{1+V^\star}\left(1 + V^\star e^{-\left(\frac{1+V^\star}{V^\star}\right)t^\star}\right),$$

$$C_{BL}^0(t^\star) = \frac{1}{1+V^\star}\left(1 - e^{-\left(\frac{1+V^\star}{V^\star}\right)t^\star}\right).$$

For $t \to \infty$, $C_B^0(t^\star \to \infty) = C_{BL}^0(t^\star \to \infty) = \frac{1}{1+V^\star}$ to be interpreted as that at times long compared to the short time scale $t_Q$ the APAP concentration in the liver and in the extrahepatic blood compartment equilibrate to a (dimensionless) concentration $\frac{1}{1+V^\star}$.

Now looking at the large time scale, $t_{PS}$, we first define another non-dimensionalization: $c_B^\star = \frac{C_B}{C_0}, c_{BL}^\star = \frac{C_{BL}}{C_0}, c_H^\star = \frac{C_H}{C_0}$ and $T^\star = \frac{t}{t_{PS}}$. This leads to

$$\epsilon \frac{dc_B^\star}{dT^\star} = (c_{BL}^\star - c_B^\star),$$

$$\epsilon V^\star \frac{dc_{BL}}{dT^\star} = -(c_{BL}^\star - c_B^\star) - \epsilon(c_{BL}^\star - c_H^\star).$$

Assuming a similar asymptotic expansion: $c_i^\star = c_i^0 + \epsilon c_i^1$ for each concentration field (we again dropped the $\star$ for readability) and gathering the terms of lowest order gives

$$c_{BL}^0(T^\star) = c_B^0(T^\star).$$

Looking at the next order

$$\frac{dc_B^0}{dT^\star} = c_{BL}^1 - c_B^1$$

$$\frac{V^\star dc_{BL}^0}{dT^\star} = -(c_{BL}^1 - c_B^1) - (c_{BL}^0 - c_H^0)$$

Using $c_{BL}^0(T^\star) = c_B^0(T^\star)$ from the lowest order result, multiplying the first equation for $dc_B^0(T^\star)/dT^*$by $(-V^*)$ and adding it to the second equation yields

$$c_{BL}^{1} - c_{B}^{1} = -\frac{1}{1+V^\star}(c_{BL}^{0} - c_{H}^{0})$$

With $c_{BL}^{0}(t) = c_{B}^{0}(t)$, plugging this back into the previous ODE finally gives

$$\frac{dc_B^0}{dT^\star} = -\frac{1}{1+V^\star}(c_B^0 - c_H^0)$$

and the compatibility condition provides that $C_B^0(t^\star \to \infty) = \frac{1}{1+V^\star} = c_B^0(T^\star = 0)$ i.e., the long time result on the short time scale matches the short time result on the long time scale.

If we now write the ODE for the total concentration at the same order using $c_{tot} = \frac{V_B c_B^0 + V_{BL} c_{BL}^0}{V_{BL}+V_B} = c_B^0 = c_{BL}^0$

$$\frac{dc_{tot}}{dT^\star} = \frac{1}{V_{BL}+V_B}\left(V_{BL}\frac{dc_{BL}^0}{dt} + V_B\frac{dc_B^0}{dt}\right) = -\frac{V_B}{V_{BL}+V_B}(c_{tot} - c_H^0)$$

Going back to original dimension-full form gives us

$$\frac{dc_{tot}}{dt} = -\frac{PS}{V_{BL}+V_B}(c_{tot} - c_H^0)$$

which is the equivalent ODE of the well-mixed model without a blood liver compartment as it was used in CL-model 3. This proves in the limit $\frac{K_{PS}}{K_Q} = \epsilon \ll 1$, the “core set of mechanisms” of the CL-4 model will be equivalent to a simplified CL-3 model. Note, that rates of feeding the blood compartment from the peritoneum, of decrease by the kidney (and other processes) as well as of intrahepatocyte degradation may define further characteristic time scales. E.g., even if $PS$ is large (so that $\frac{PS}{Q_{liver}}$ is not small anymore, a very slow intrahepatocyte degradation process of APAP may render the flux of APAP into the hepatocyte small. Moreover, the hepatocytes die during the intoxication hence the APAP sinks drop. Hence the study of the full dynamics requires the simulation of the entire process.

Nevertheless, the upper calculation gives a hint towards a qualitative understanding of why CL-model 3 and a spatially refined model with more spatial compartment, as the ST-model, may differ in their results, if all parameters of CL-model 3 are kept in the spatially refined (here: ST-) model.

A simple extension of that CL-model 4 would be to take into account the difference of the hepatocytes in the lobule by not considering an “average” hepatocyte.

In this case the calculation becomes very similar as can be seen by considering for the liver blood compartment instead the equation:

$$\frac{dC_{BL}}{dt} = -\frac{Q_{liver}}{V_{BL}}(C_{BL} - C_B) - \frac{PS_{liver}}{V_{BL}}\left(C_{BL} - \frac{1}{N_{liver}}\sum_{i}^{N_{liver}} C_H^i\right),$$

where $N_{liver}$ denotes the number of cells in the liver, and $S_{liver} = \sum_{i=0}^{N_{liver}} S_i$ the area of cells in the liver, where in addition $S_i$ has been assumed to be the same for each cell $i$.

If the smallest well-mixed compartment is the lobule with lobules organized in parallel instead, the relevant equation becomes

$$\frac{dC_{BL}}{dt} = -\frac{Q_{lobule}}{V_{B,lobule}}(C_{BL} - C_B) - \frac{PS_{lobule}}{V_{B,Lobule}}\left(C_{BL} - \frac{1}{N_{sim}}\sum_i^{N_{sim}} C_H^i\right),$$

where $N_{sim}$ denotes the number of cells simulated in the lobule (or lobule piece in case of our simulations), and $S_{lobule} = \sum_{i=0}^{N_{sim}} S_i$ the area of cells in the lobule (piece), where in addition $S_i$ has been assumed to be the same for each cell $i$. $V_{B,lobule}$ denotes the volume of blood in the lobule.

Consider now the parameters in our model simulations: At the individual hepatocyte level, considering an exchange surface as described in SI section 1.6.2 of $S = 4300\ \mu m^2$, the permeability fitted with CL-3 model of $P = 2.3\ \mu m/s$, the average flow rate in a sinusoid of a liver lobule piece of $\bar{Q}_{sin} = 3866\ \mu m^3/s$, one obtains a ratio $\frac{P\,S}{\bar{Q}_{sin}} \approx 2.56 > 1$. At the liver lobule piece level, considering the total exchange surface of the hepatocytes of a liver lobule piece, $S_{lobule} = 1.38 \cdot 10^7\ \mu m^2$, the same hepatocyte permeability, $P = 2.3\ \mu m/s$ and input lobule flow rate of $Q_{lob,in} = 1.2 \cdot 10^6\ \mu m^3/s$, this gives a ratio of $\frac{PS_{lobule}}{Q_{lob,in}} \approx 26.45 \gg 1$. Note that the ratio is the same at the whole liver level since $Q_{liver} = N_{lobule} \cdot Q_{lob,in}$ and $S_{liver} = N_{lobule} \cdot S_{lobule}$.

Whenever the blood vessels are organized in parallel, the flow and total interface scale in the same way. The ratio of $\frac{Q_{lob,in}}{\bar{Q}_{sin}} = 310$ indicates that considering space with the liver lobule micro-architecture can roughly be approximated by having 310 sinusoids in parallel. Since there are 3215 hepatocytes in the lobule piece and approximately 10 hepatocytes in the PV-CV axis, this is consistent with considering 10 cells for each of those 310 sinusoids. Plugging this into the ratio of interest gives $\frac{PS_{sin}}{\bar{Q}_{sin}} = \frac{10PS}{\bar{Q}_{sin}} = 25.6$, which is similar to the other estimates derived for the lobule level and liver level. Note that this is a rough estimate as we only considered the average sinusoidal volume flow rate which can vary from one sinusoid to another.

Both of those values, $\frac{P\,S}{\bar{Q}_{sin}}, \frac{P\,S_{lobule}}{Q_{lob,in}}$ are much larger than what would be needed to justify the equivalence between the two modeling approaches in the CL-model 3 and ST-model, so from this line of argument it is not surprising if the spatially refined resolution of the ST-model compared to the CL-model 3 matters, independently of the zonation effects.

## 1.7 Model-type-components in short

**CL-model 1**: Equations in sections 1.1 – 1.3.

**CL-models 2 & 3**: Equations in section 1.1, 1.2, 1.4; for model version with space of Disse (considered in Fig. 5): section 1.5 instead of section 1.4. CL-model 2 (without the space of Disse) and CL-model 3 differ only by the way they are parameterized.

**ST-model**: Equations in sections 1.1, 1.2, 1.6.1 – 1.6.3.

For initial values of the intracellular model, see section 1.9. Initial values for ST model see section 1.6.4.

## 1.8 Calibration of parameters

For a given set of measures $\hat{y}_i$ and corresponding simulations at times $t_i$ for a vector of parameters $\theta$, the likelihood function quantifies the agreement between the data points and the model simulations. To have the best agreement between data and model simulations, this function has to be maximized. I n practice one minimizes a cost function based off the log-likelihood function. This reads as:

$$C(\theta) = -2\ln(L) = \sum_{i=1}^{n} \frac{\left(\hat{y}_i - y(t_i, \theta)\right)^2}{\sigma_i^2}$$

Where $\sigma_i$ are the standard deviations of the data considered and $n$ the total number of data points. This cost-function is then minimized using the Covariance matrix adaptation evolution strategy (CMA-ES) (Hansen and Ostermeier, 1996; Hansen, 2006) which is a well-established global convergence algorithm for non-linear functions.

### 1.8.1 Cost functions expressions

For *in vitro* toxicity data, the cost function reads as

$$C_{vitro}(\theta_{intracell}) = \sum_{i=1}^{6} \frac{\left(\hat{y}_i - y(t = 24h, \theta_{intracell}, C_i^0)\right)^2}{\sigma_i^2} + \sum_{1}^{5} \frac{\left(\hat{y}_i - y(t_i, \theta_{intracell}, C^0 = 4mM)\right)^2}{\sigma_i^2}$$

With $C_i^0$ the initial concentration in the medium, $\hat{y}_i$ the experimental fraction of dead cells, $y(t, \theta_{intracell}, C^0)$ the fraction of dead cells as simulated by the *in vitro*-PD model of APAP intracellular metabolism at time $t$ for a set of intracellular parameters $\theta_{intracell}$ and an initial medium concentration $C^0$. This quantifies the agreement of the model to both available data sets: at 24h for several concentrations (first sum) and over time for a single concentration (second sum).

For the *in vivo* APAP blood concentration, the cost function reads as:

$$C_{vivo,blood}(\theta_{extracell}) = \sum_{i=1}^{4} \frac{\left([\widehat{APAP}]_i - [APAP](t = 30min, \theta_{extracell}, D_i^0)\right)^2}{\sigma_i^2} + \sum_{1}^{7} \frac{\left([\widehat{APAP}]_i - [APAP](t_i, \theta_{extracell}, D^0 = 300mg/kg)\right)^2}{\sigma_i^2}$$

With $D_i^0$ the initial dose, $[\widehat{APAP}]_i$ the experimental concentration of APAP in blood, $[APAP](t, \theta_{extracell}, D^0)$ the concentration of APAP in blood as simulated the different strategies studied (PK model, or coupled model) at time $t$ for a set of intracellular parameters $\theta_{extracell}$ and an initial dose $D^0$. This quantifies the agreement of the model to both available data sets: at 30min for several doses (first sum) and over time for a single dose (second sum). Note that when the model to

calibrate is the coupled one, the concentration of APAP also depends on $\theta_{intracell}$ for which the CYP $V_{max}$ and the initial GSH concentration are corrected by their respective factors (see SI section 1.2).

Depending on the fit strategy used, an additional cost function for a single data point can be used *in vivo* to constrain the parameters such that the elimination of APAP by the liver is not small compared to the elimination of other organs modelled as $k_{others}n_b$. The target value of elimination from the liver is 85% of the initial bioavailable dose $F \cdot n_0$ (Prescott and Wright, 1973; Dai et al., 2006; Malfatti et al., 2020). The tolerance to reach this target is set in the "standard deviation" as 20%. The cost function thus reads as

$$C_{vivo,elim}(\theta_{extracell}) = \frac{(0.85 \cdot F \cdot n_0 - Lu(t = 480min, \theta_{extracell}, D^0 = 300mg/kg))^2}{(0.2 \cdot F \cdot n_0)^2}$$

With $Lu(t = 480min, \theta_{extracell}, D^0 = 300mg/kg)$ the total liver uptake at time $t = 480min$ for a given set of extracellular parameters, for a given dose of $D^0 = 300mg/kg$. This reads as

$$Lu = PS \frac{N_{liver}}{N_{lob}} \sum_{i=1}^{N_{lob}} \int_{t=0}^{480min} ([APAP]_{blood} - [APAP]_{cell}^{i})$$

Where $[APAP]_{blood}$ and $[APAP]_{cell}^{i}$ are the simulated concentration of blood and in cell with index $i$ for a 300mg/kg dose. $Lu$ thus quantifies the total amount of moles entering the hepatocytes of the liver over a period of 480 minutes.

### 1.8.2 Cost function per strategy

Here after is a summary of the cost functions used for each of the main strategies developed in the main text:

- Str2: Minimize $C_{vitro}(\theta_{intracell})$ using *in vitro* model and independently minimize $C_{vivo,blood}(\theta_{extracell})$ using the PK model (CL-model 1, Fig. 2A2).Str3: Minimize $C_{vitro}(\theta_{intracell})$ using *in vitro* model, as a second step minimize $C_{vivo,blood}(\theta_{extracell}) + C_{vivo,elim}(\theta_{extracell})$ using the multi-compartment model with $\theta_{vitro}$ fixed as fitted in the first step (CL-model 2, Fig. 2B2).
- Str4: Minimize simultaneously $C_{vitro}(\theta_{intracell}) + C_{vivo,blood}(\theta_{extracell}) + C_{vivo,elim}(\theta_{extracell})$ using the multi-compartment model (CL-model 3, Fig. 2C1).

## 1.9 Initial parameter values

Literature research was performed to identify initial guess and ranges of parameters used in the metabolic model. Table 1 indicates the references used for the initial parameter values as well as the unit and species.

For the extra-cellular parameters involved in the *in vivo* PD-compartment model, initial values from the PK models were used.

| Name | Literature value | Initial value | Unit | Reference | Species |
|---|---|---|---|---|---|
| $P$ | 30 | 30 | µm/min | (Irvine et al., 1999) | Cell line |
| $V_{max,CYP2E1}$ | 6, 23 | 13 | µM/min | (Hu et al., 1993; Patten et al., 1993) | Mice, rat |
| $K^{m}_{CYP2E1}$ | 30, 43-45 | 45 | µM | (Hu et al., 1993; Patten et al., 1993) | Mice, rat |
| $V_{max,CYP1A2}$ | 105, 433 | 242 | µM/min | (Hu et al., 1993; Patten et al., 1993) | Mice, rat |
| $K^{m}_{CYP1A2}$ | 870, 600-700 | 700 | µM | (Hu et al., 1993; Patten et al., 1993) | Mice, rat |
| $V_{max,SULT}$ | 7, 170, 4.5, 150, 27, 22 | 50 | µM/min | (Mizuma et al., 1985; Miller and Jollow, 1987; Iida et al., 1989; Studenberg and Brouwer, 1993; Liu and Klaassen, 1996; Riches et al., 2009) | Rat, hamster, rat, rat, mice, human |
| $K^{m}_{SULT,APAP}$ | 480,30, 300, 2000, 30 | 300 | µM | (Sekura and Jakoby, 1979; Mizuma et al., 1985; Miller and Jollow, 1987; Iida et al., 1989; Riches et al., 2009) | Rat, rat, hamster, rat, human |
| $K^{m}_{SULT,PAPS}$ | 5, 10 | 10 | µM | (Sekura and Jakoby, 1979; Nagar et al., 2006) | Rat, human |
| $V_{max,UGT}$ | 33, 200, 56, 200, 15 | 100 | µM/min | (Mizuma et al., 1985; Iida et al., 1989; Miners et al., 1990; Studenberg and Brouwer, 1993; Riches et al., 2009) | Rat, rat, human, rat, human |
| $K^{m}_{UGT,APAP}$ | 700, 2100, 6000, 2000 | 2000 | µM | (Mizuma et al., 1985; Iida et al., 1989; Miners et al., 1990; Riches et al., 2009) | Rat, rat, human, human |
| $K^{m}_{UGT,UDPg}$ | 680 | 460 | µM | (Miners et al., 1990) | Human |

| | | | | | |
|---|---|---|---|---|---|
| $V_{max,GST}$ | - | 1000 | µM/min | - | - |
| $K^{m}_{GST,NAPQI}$ | 7 | 7 | µM | (Coles et al., 1988) | Rat |
| $K^{m}_{GST,GSH}$ | 300-5000,<br>180 | 200 | µM | (Kraus, 1980; Phillips and Mantle, 1991; Pezzola et al., 2010) | Rat, mice, human |
| $k_{on}$ | 0.0016 | 0.016 | 1/min | (Howell et al., 2012) | Rat |
| $n1$ | - | 5 | - | - | - |
| $n2$ | - | 3 | - | - | - |
| $k_{p,UDPg}$ | 100 | 100 | µM/min | (Bock and White, 1974; Reinke et al., 1981; Conway et al., 1985) | Rat, rat, rat |
| $k_{d,UDPg}$ | 0.3 | $k_{p,UDPg}$ $/300$ | 1/min | (Bock and White, 1974; Zhivkov et al., 1975; Singh and Schwarz, 1981; Hjelle et al., 1985) | Rat, rat, rat, rat |
| $k_{p,PAPS}$ | 50 | 50 | µM/min | (Reinke et al., 1981; Sweeny and Reinke, 1988; Dalhoff and Poulsen, 1992; Kim et al., 1995) | Rat, rat, rat, rat |
| $k_{d,PAPS}$ | 3 | $k_{d,PAPS}$ $/150$ | 1/min | (Reinke et al., 1981; Sweeny and Reinke, 1988; Dalhoff and Poulsen, 1992; Kim et al., 1995) | Rat, rat, rat, rat |
| $k_{p,GSH}$ | 8.4 | 8.4 | µM/min | (Akerboom et al., 1982; Huang et al., 1998) | Rat, rat |
| $k_{d,GSH}$ | 0.028 | $k_{p,GSH}$ $/12757.5$ | 1/min | (Akerboom et al., 1982; Huang et al., 1998) | Rat, rat |
| $V_{max,ROSp}$ | - | 1000 | µM/min | - | - |

| | | | | | |
|---|---|---|---|---|---|
| $K^{m}_{ROSp}$ | - | 100 | µM | - | - |
| $V_{max,ROSd}$ | - | 500 | µM/min | - | - |
| $K^{m}_{ROSd,ROS}$ | - | 1000 | µM | - | - |
| $K^{m}_{ROSd,GSH}$ | - | 1000 | µM | - | - |
| $k_{p,Mito}$ | - | 0.01 | µM/min | - | - |
| $k_{d,Mito}$ | - | $k_{p,Mito}/1$ | 1/min | - | - |
| $V_{max,MPT}$ | - | 10 | µM/min | - | - |
| $K^{m}_{MPT}$ | - | 1000 | µM | - | - |
| $k_{p,ATP}$ | 500000 | 500000 | µM/min | $[ATP]_0 = 5000\mu M$. (Drew and Leeuwenburgh, 2003) | Rat |
| $k_{d,ATP}$ | 100 | $k_{p,APAP}$ $/500000$ | 1/min | (Drew and Leeuwenburgh, 2003) | Rat |
| $CV$ | 0.25 | 0.25 | - | (Sigal et al., 2006; Spencer et al., 2009) | Human, human |

## 1.10 Preliminary sensitivity analysis

Before performing optimization of the cost-function, a sensitivity analysis using the *in vitro* compartment model was done by simply varying each parameter by a factor ranging from 1/100 to 100 while keeping the other constants for a concentration of 500µM. This permitted to identify the parameters that have almost no effect on the cost-function and those values were thus kept constant and not calibrated during the optimization step.

Table 2 here after displays the parameters that were identified as sensitive and the ranges on which they will be fitted. Their initial value is set based on the column “Initial value” of Table 1. All other parameters are kept constant to the “Initial Value” column of Table 1.

| **Name** | **Ranges** |
|---|---|

| | |
|---|---|
| $P$ | $\left[\frac{30}{10}, 30 \times 10\right]$ |
| $V_{max,CYP2E1}$ | $\left[\frac{23}{50}, 23 \times 10\right]$ |
| $K^m_{CYP2E1}$ | $\left[\frac{43}{5}, 45 \times 3\right]$ |
| $V_{max,CYP1A2}$ | $\left[\frac{433}{50}, 433 \times 10\right]$ |
| $K^m_{CYP1A2}$ | $\left[\frac{600}{3}, 700 \times 5\right]$ |
| $V_{max,GST}$ | $\left[\frac{1000}{100}, 1000 \times 100\right]$ |
| $K^m_{GST,GSH}$ | $\left[\frac{200}{100}, 200 \times 100\right]$ |
| $k_{on}$ | $\left[\frac{0.0016}{100}, 0.0016 \times 100\right]$ |
| $n1$ | [3.5,7.5] |
| $k_{d,GSH}$ | $\left[\frac{0.0028}{100}, 0.0028 \times 1000\right]$ |
| $V_{max,ROSp}$ | $\left[\frac{1000}{100}, 1000 \times 100\right]$ |
| $K^m_{ROSp}$ | $\left[\frac{100}{100}, 100 \times 100\right]$ |
| $V_{max,ROSd}$ | $\left[\frac{500}{100}, 500 \times 100\right]$ |
| $k_{p,Mito}$ | $\left[\frac{0.01}{100}, 0.01 \times 100\right]$ |

| | |
|---|---|
| $V_{max,MPT}$ | $\left[\frac{1}{100}, 1 \times 1000\right]$ |
| $K^{m}_{MPT}$ | $\left[\frac{1000}{1000}, 1000 \times 1000\right]$ |
| $CV$ | $[0.05, 0.5]$ |

## 1.11 Estimation of CYP positive areas and enzyme activity gradient

To determine the fraction of CYP2E1-positive and CYP1A2-positive cells, a manual segmentation of immunostained mice liver slices was performed (for each CYP, 4 images in 4x magnification containing in total around 60 lobules) (SFig. 5A, B). This indicated $50.9\% \pm 0.9$ of CYP2E1-positive cells and $60.5\% \pm 3.6$ of CYP1A2 positive cells (Cellière, 2016).To determine the CYP enzyme activity, a gradient blur was performed on the CYP-immunostained images and the intensity is quantified along the porto-central axis (indicated by the red line in SFig. 5C). This produces a roughly linearly decreasing profile. (SFig. 5D). We assume that the intensity variation translates as an enzyme activity variation. For CYP2E1, this means that the value is maximal closest to the central vein and only half of it at the border of the CYP2E1 positive area. For CYP1A2, the value is maximal closest to the central value and drops to 2/3 at the border of the CYP1A2 positive area.

## 1.12 A posteriori sensitivity analysis

We performed a sensitivity analysis similar to the one described in section 1.9 around the set of parameters identified in Fig. 6 of the main text i.e., each parameter that had been fitted using strategy 4 (Fig. 6) was varied individually while the other parameters were kept constant. SFig. 8 displays the fraction of dead cells *in vitro* for a single initial concentration of 4mM and different scaling factors of parameters. This concentration was chosen as this is the one for which time course data was available. The reference case corresponds to scaling factor equal to 1, i.e. corresponding to the result in Fig. 6. If, for a given parameter scaling factor, the relative difference of the fitness function to the reference case was less than 50%, a simulation in the *in vivo* setting was performed for a single dose of 300mg/kg and the corresponding scaling factor of that parameter to study if the *in vivo* simulation result may be more affected by the change of that parameter than the *in vitro*-result (SFig. 9). This dose was chosen as time course data is available for the APAP blood concentration and one can safely assume that the associated damage will be close to the 281mg/kg's dose for which the damage is similar to the 4mM concentration *in vitro*. Five parameters were identified as non-sensitive *in vitro*: the permeability, $V_{max,CYP2E1}$, $K^{m}_{CYP2E1}$, $K^{m}_{GST,GSH}$ and $n1$ . Changing the same parameters by the same amount in the simulation for the *in vivo* toxicity also lead to small (largely negligible) effect on the APAP blood concentration and fraction of dead cells (SI Fig. 9). Increasing the permeability did not have an effect on the fraction of dead cells but lowering it by a factor 100 did. This can be interpreted as if the permeability parameter fitted originally was already on the range of high values which make the system not limited by permeability. Thus, increasing its value does not impact the simulation while lowering it significantly does. Surprisingly, the model is not sensitive to the CYP2E1 enzyme reaction parameters. More precisely, multiplying the maximum velocity of the CYP2E1 enzyme did have a sensitive impact but only moderate changes and even lowering it by a

factor 100 did not impact the fraction of dead cells significantly. This means that in this parameter fit set, the damage originates primarily from the CYP1A2 enzyme. This can also be explained by three other aspects (table 1): (i) the initial value of the CYP2E1 is lower than the CYP1A2, (ii) the fraction of cells which are CYP1A2 is 10% more than the CYP2E1 positive cells (section 1.11), (iii) the gradient of the CYP1A2 only drops to 2/3 of its maximum value, while it drops to half of its maximum value in the case of CYP2E1 (section 1.11). Finally, both $K^{m}_{GST,GSH}$ and the Hill coefficient of ROS production $n1$ were identified as not sensitive parameters. However, the other parameters involved in the GST enzyme reaction and ROS productions were identified as sensitive parameters. Moreover, it is possible that proper groupings of those sensitive parameters are again insensitive.

# 2 Supplementary figures

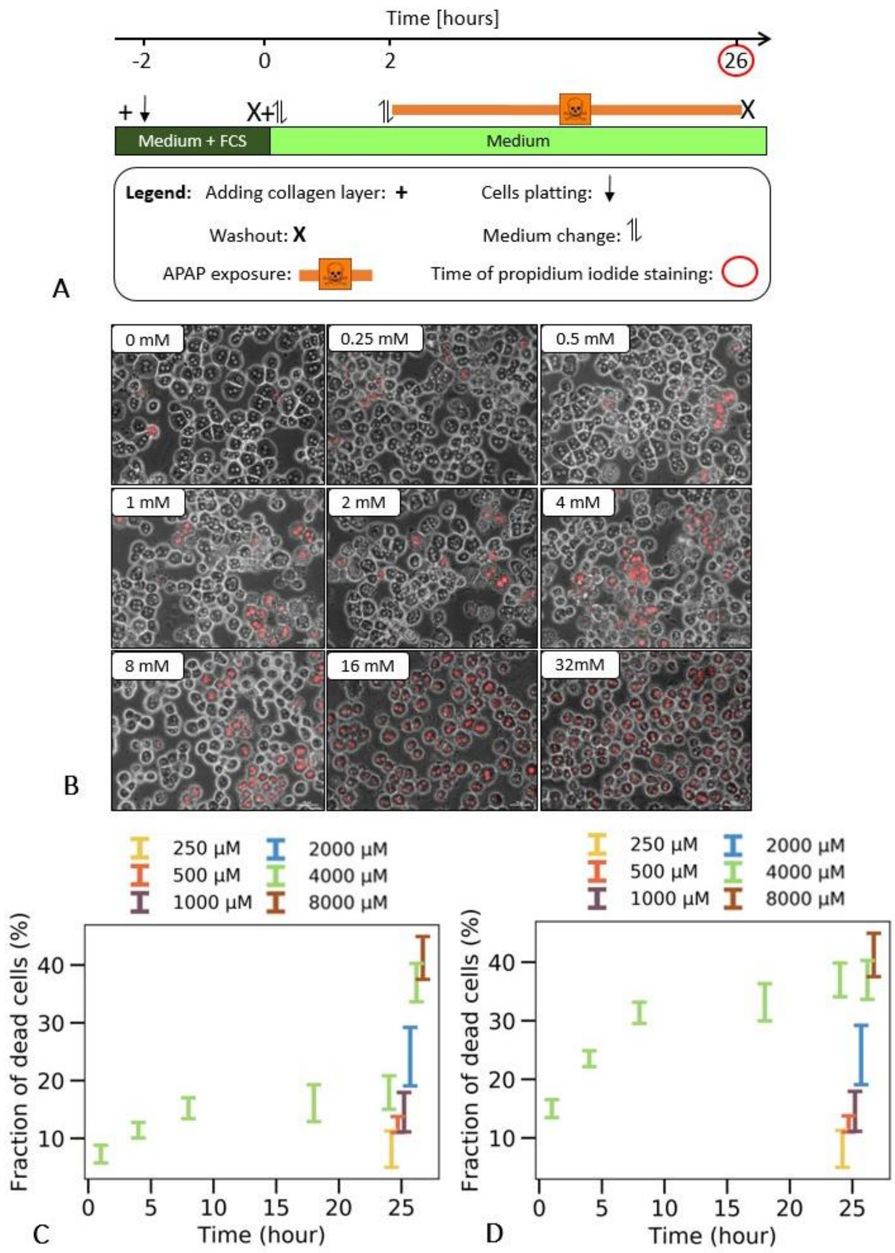


**Supplementary Figure 1. *In vitro* experimental determination of APAP hepatotoxicity.** Experimental design (A), representative snapshots of hepatocytes at 24 hours after incubation with different concentrations of APAP (B), fraction of dead cells over time of original data (C) and after rescaling (D). The six 24h-values have been determined from cultivated hepatocytes of the same mouse exposed to six different APAP concentrations. The kinetics (left blue bars in (C)) was determined from another mouse. The rescaling was required as the precise toxicity value at 24h

depends on the GSH level of the respective mouse before starvation, that cannot be precisely controlled.

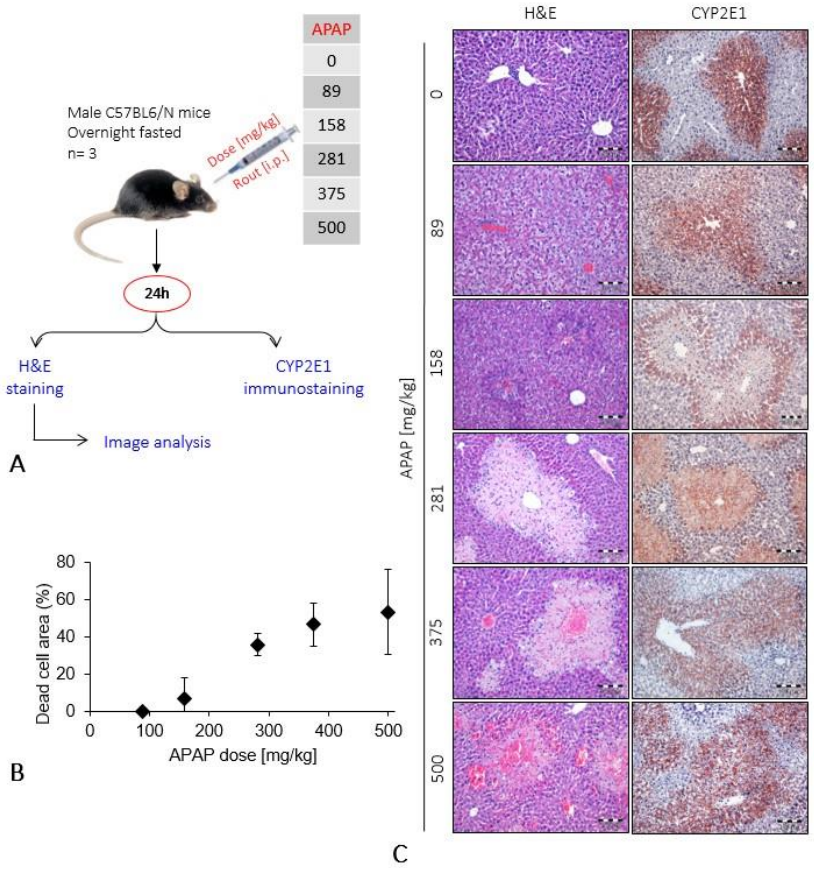


**Supplementary Figure 2. *In vivo* experimental determination of APAP hepatotoxicity.** Procedure (A), fraction of dead cells versus dose (B), snapshots of H&E staining (left) and CYP2E1 (right) for different doses (C).

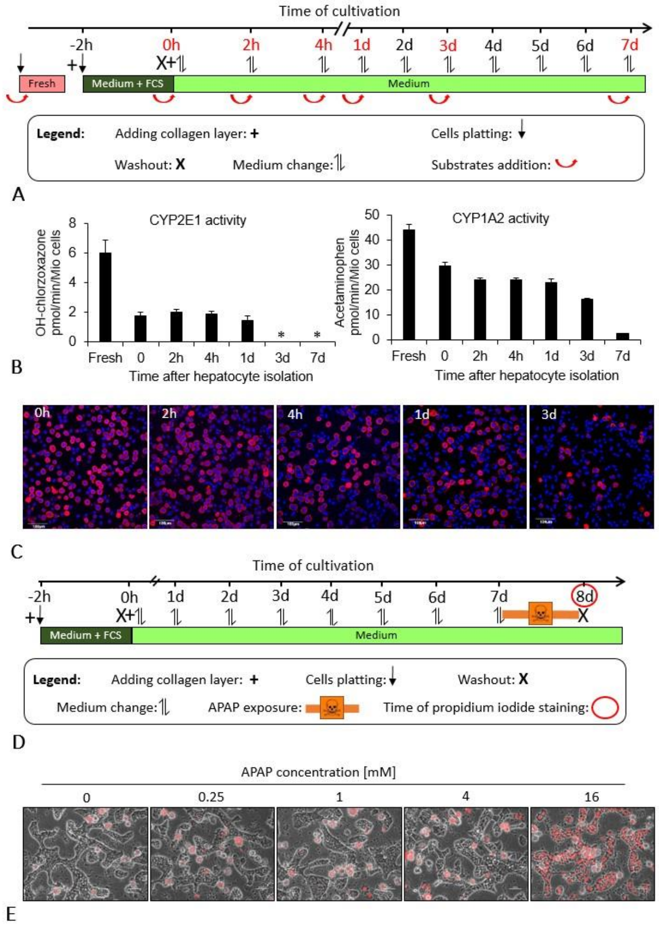


**Supplementary Figure 3. Time-dependent loss of Cyp450 enzyme activity after hepatocyte isolation.** A. Experimental design. B. Cyp2e1 and Cyp1a2 activity assay in isolated microsomes at different time intervals after hepatocyte isolation. C. Cyp2e1 immunostaining at different time

intervals after hepatocyte isolation and cultivation. D, E. Cytotoxicity of APAP on cultivated hepatocytes after incubation on day 7 after isolation.

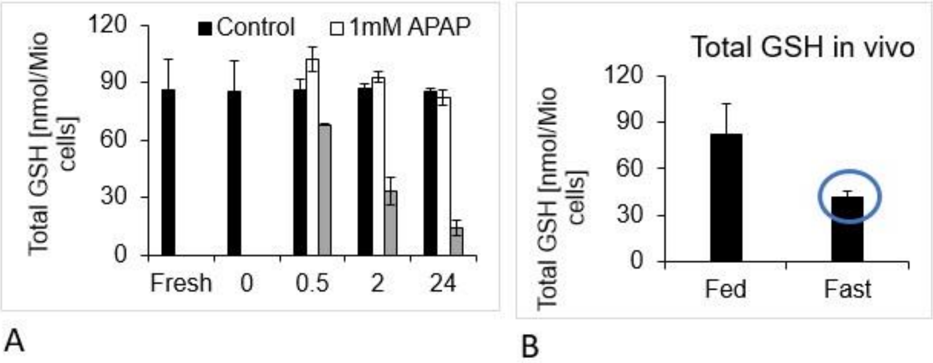


**Supplementary Figure 4.** Total GSH (nmol/Mio cells) *in vitro* (A), and *in vivo* (B).

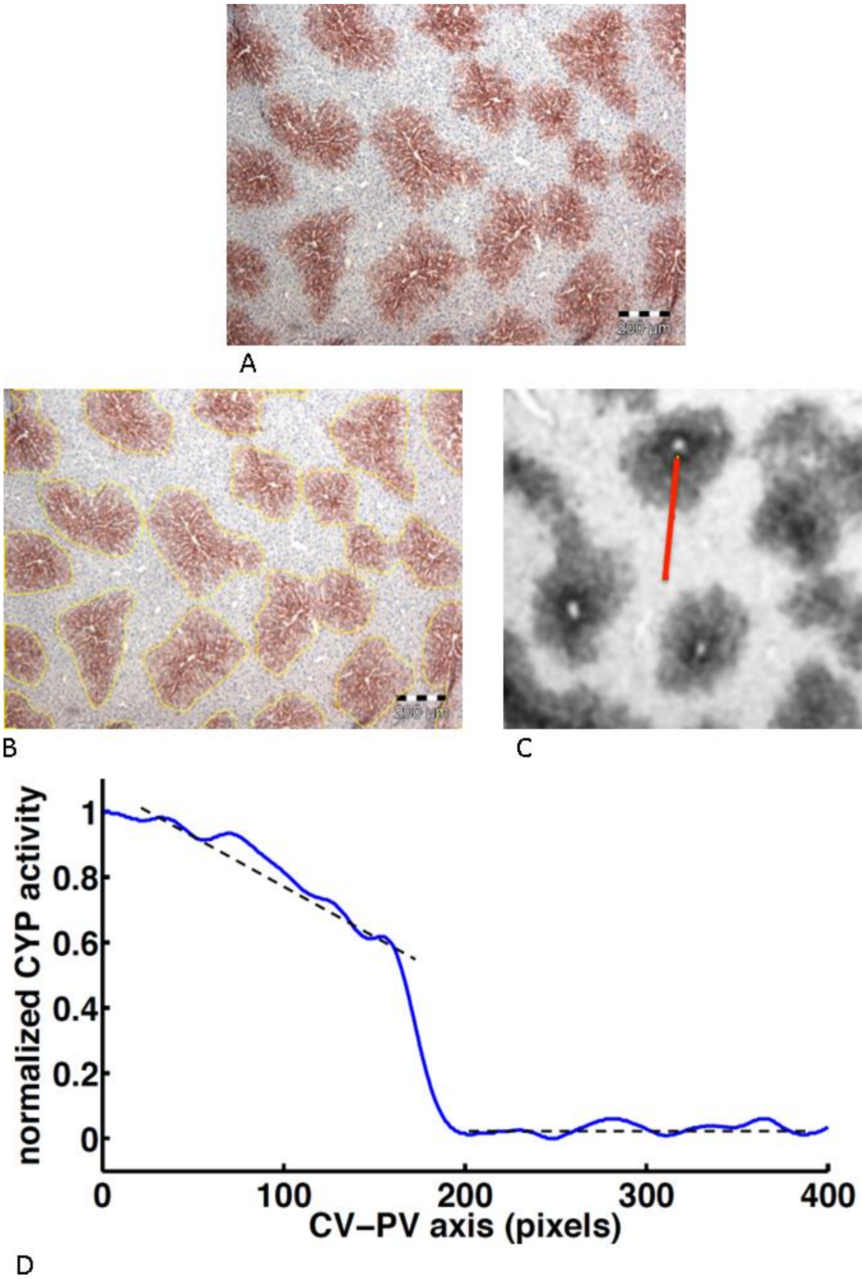


**Supplementary Figure 5. Image processing workflow for CYP area and gradient quantification.** (A) CYP2E1 staining, (B) manual segmentation of the CYP2E1 positive areas (yellow lines), (C) gradient blur result and PV-CV axis (read line), (D) normalized CYP2E1 activity.

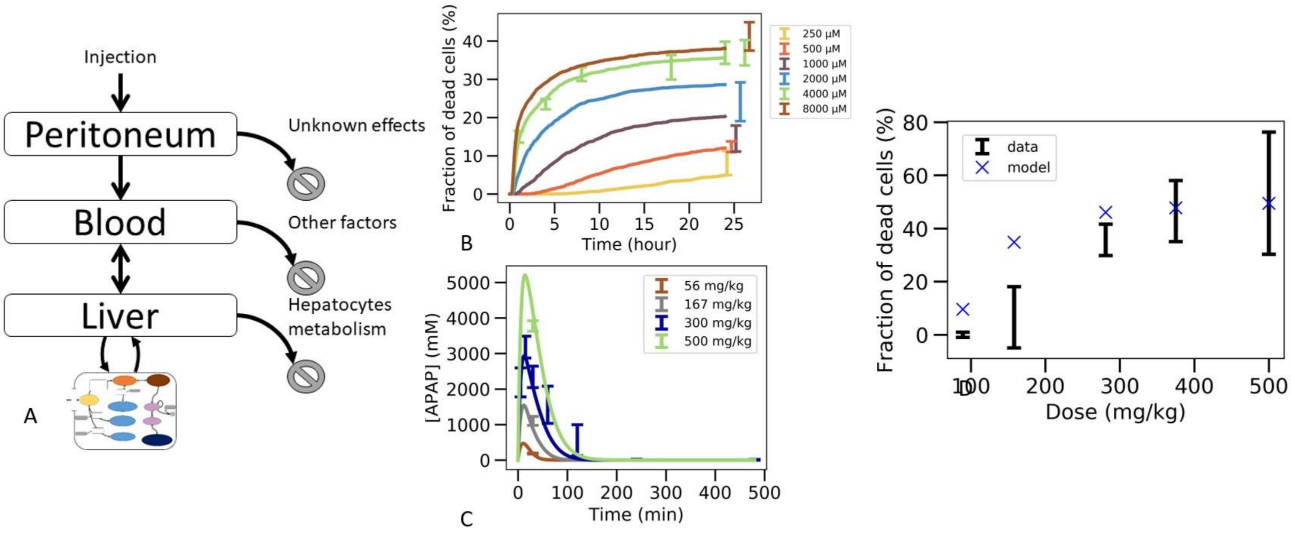


**Supplementary Figure 6. Second simultaneous fit to *in vitro* toxicity data and *in vivo* pharmacokinetic data strategy to test sensitivity to parameter sets. (**A) Scheme of the model, (B)

fit to *in vitro* toxicity data, (C) fit to *in vivo* pharmacokinetic data, (D) extrapolation to *in vivo* toxicity data.

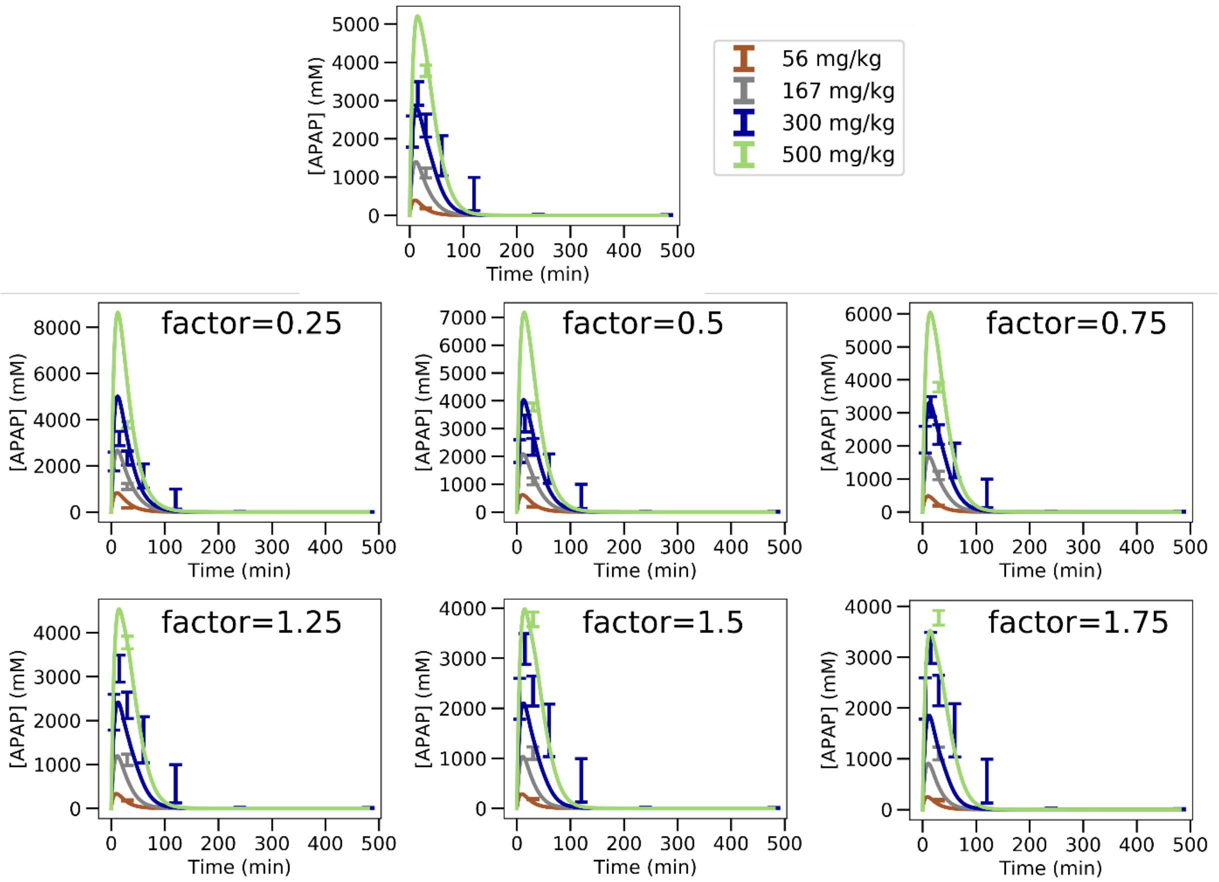
[APAP] (mM)
Time (min)
56 mg/kg
167 mg/kg
300 mg/kg
500 mg/kg
factor=0.25
factor=0.5
factor=0.75
factor=1.25
factor=1.5
factor=1.75

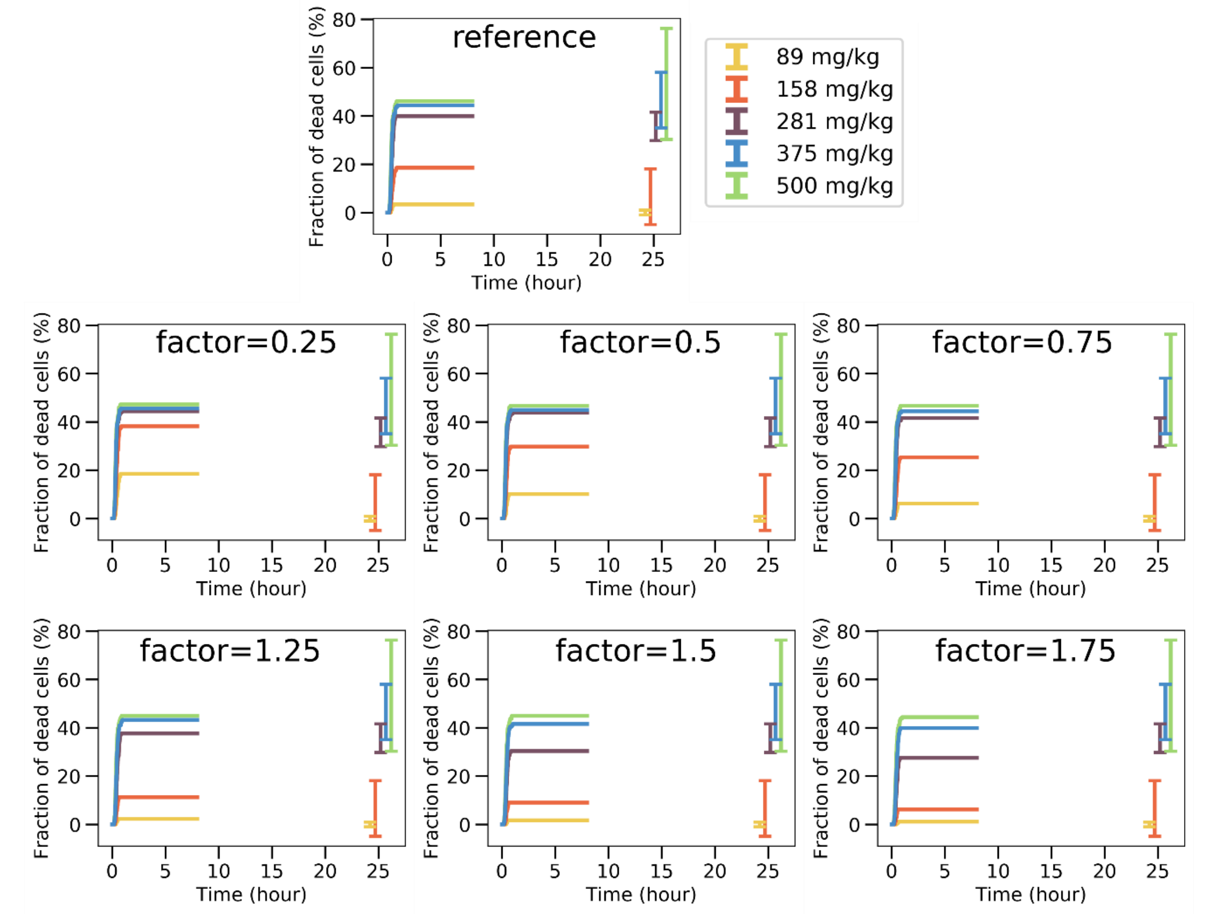
Fraction of dead cells (%)
Time (hour)
reference
89 mg/kg
158 mg/kg
281 mg/kg
375 mg/kg
500 mg/kg
factor=0.25
factor=0.5
factor=0.75
factor=1.25
factor=1.5
factor=1.75

**Supplementary Figure 7. Influence of $N_{liver}$ on APAP blood concentration and fraction of dead cells *in vivo* in the CL-3 model.** A scaling factor is applied on the number of cells in the liver on the best fit obtained for the CL-3 model. Top row: reference for APAP blood concentration (i.e. scaling factor of 1), second and third rows: APAP blood concentration for scaling factors from 0.25 to 1.75, fourth row: reference for fraction of dead cells (i.e. scaling factor of 1), fifth and sixth rows: fraction of dead cells for scaling factors from 0.25 to 1.75.

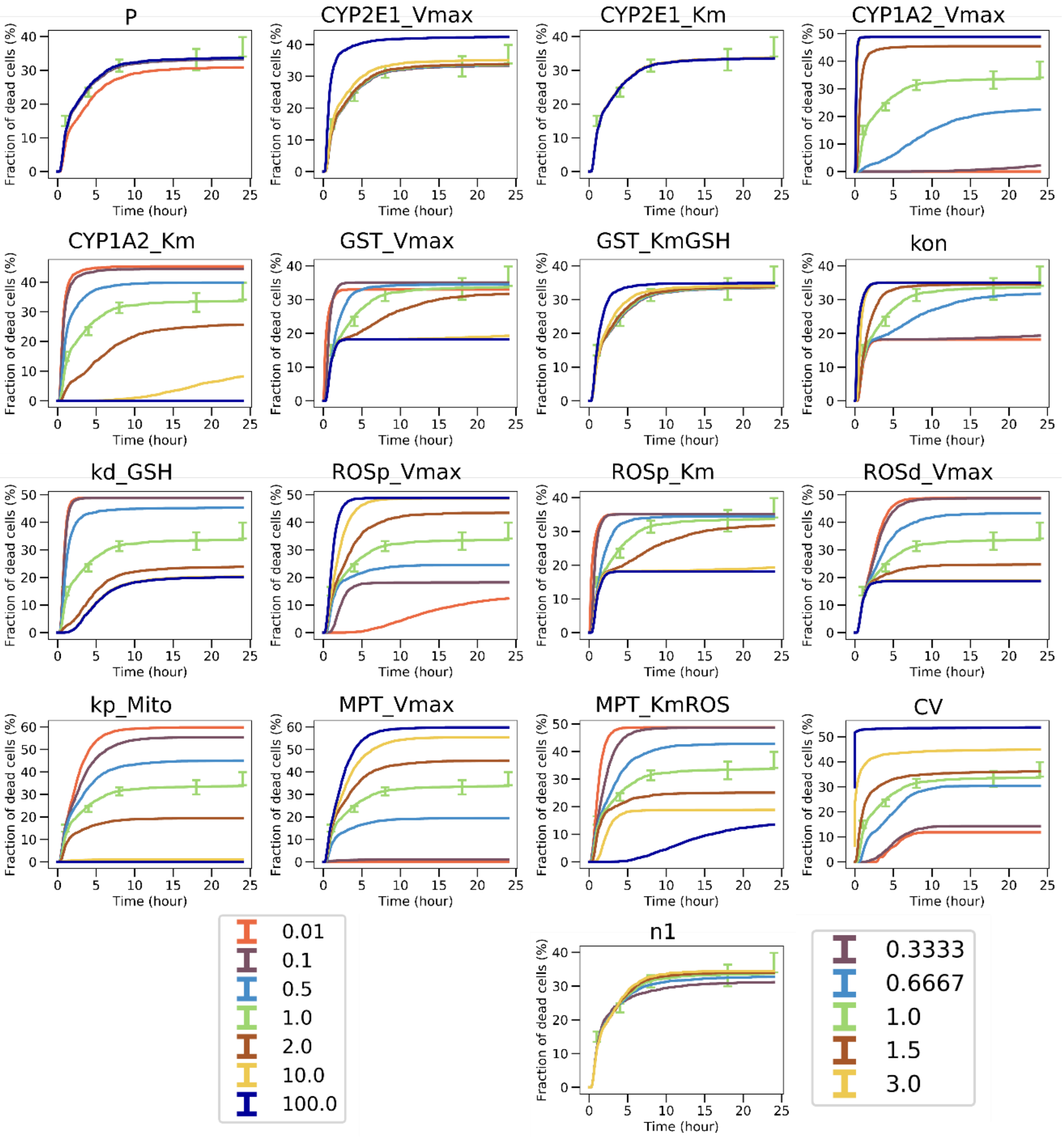


**Supplementary Figure 8. Sensitivity analysis *in vitro* around the parameter set that allows the best fit to available data and best extrapolation to *in vivo* toxicity data**. The panels represent the fraction of dead cells over time for a single initial concentration of 4mM for all parameters that were identified as sensitive ones in the preliminary step (section 1.9). Bottom left legend corresponds to the 16 first panels. A different scaling for the n1 parameter is used as it represents a power. Bottom right legend indicates the scaling for this parameter.

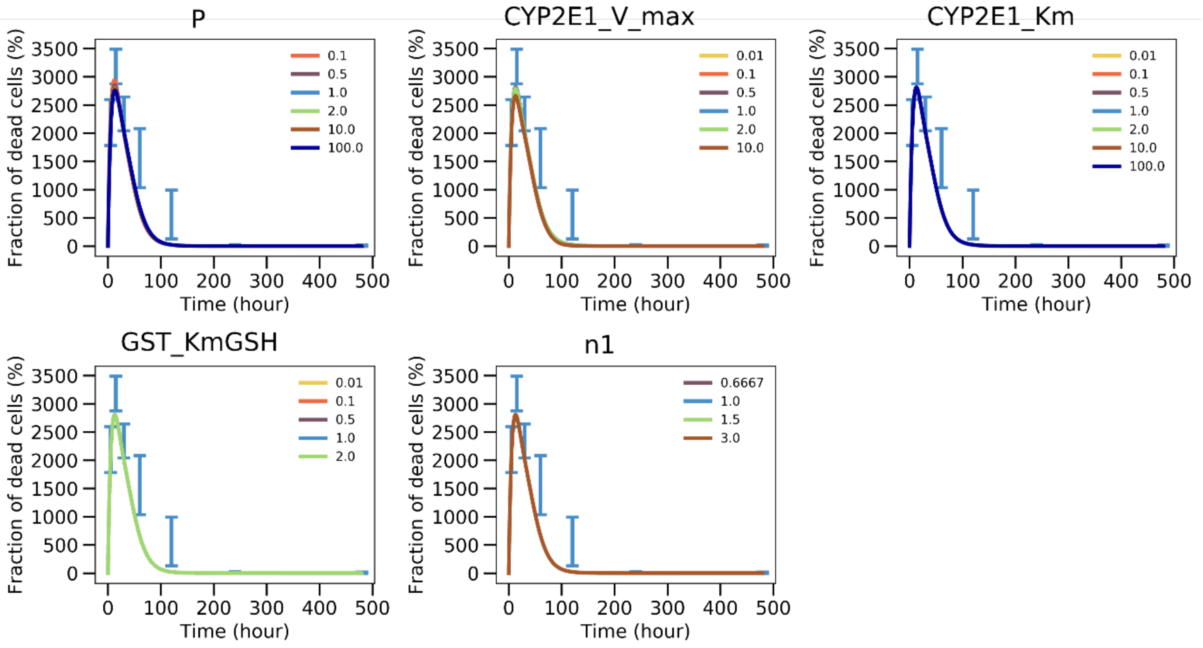


**Supplementary Figure 9. Sensitivity analysis *in vivo* for non-sensitive parameters' scalings.** The panels represent the blood concentration for a 300mg/kg dose for the parameters identified as not sensitive *in vitro* (Fig. 8).

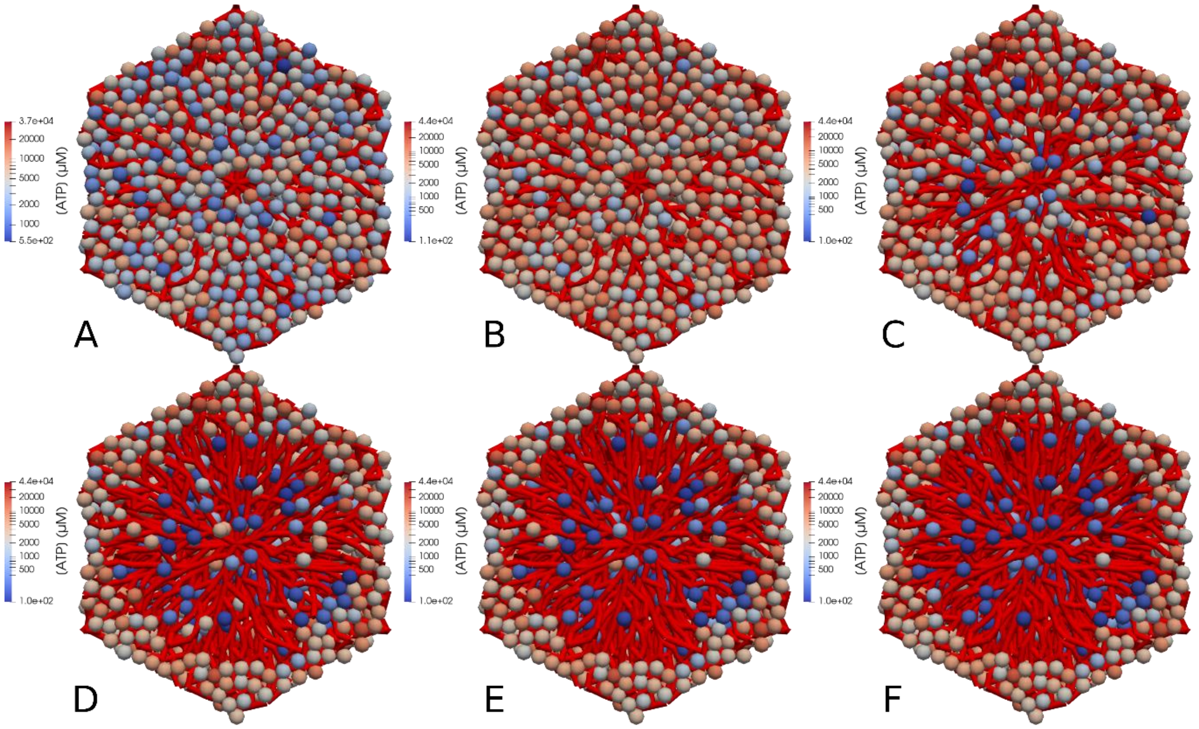


**Supplementary Figure 10. Comparison of final spatial damage for all doses for which hepatotoxic data *in vivo* is available.** (A) Control (mimicked as initial state for a 89mg/kg, i.e. when no damage occurred). Spatial damage at 24h (extrapolated from the steady state of the fraction of dead cells in simulations, see Fig. 7C), which we extrapolate as final damage at 24h for doses (B) 89 mg/kg (C) 158 mg/kg, (D) 281 mg/kg, (E) 375 mg/kg, (F) 500 mg/kg.

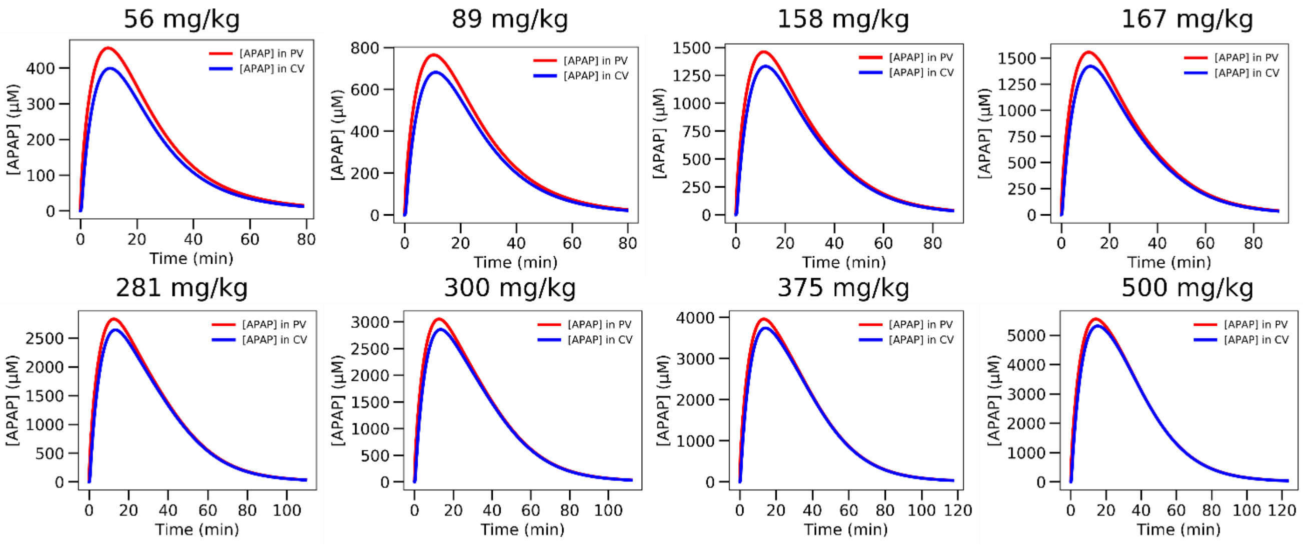


**Supplementary Figure 11. APAP concentration in portal vein (red) and central vein (blue) for all doses for which simulations were ran.** The same parameters than on Fig. 7 were used.

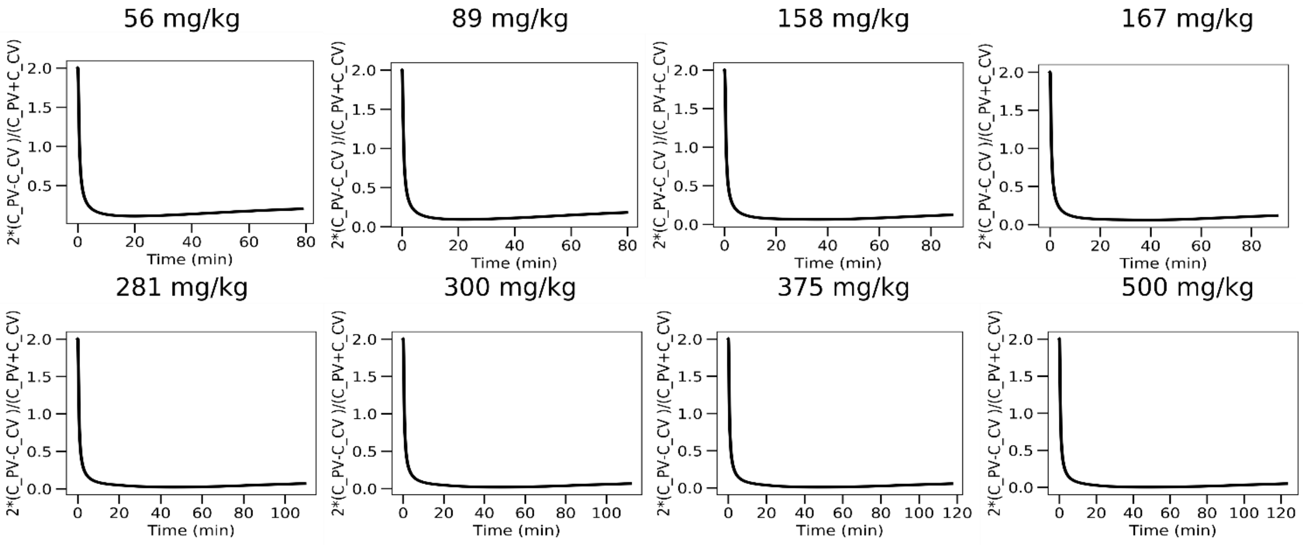


**Supplementary Figure 12.** Relative change of APAP concentration between portal vein and central vein $\frac{(C_{PV}-C_{CV})}{\frac{1}{2}(C_{CV}+C_{PV})}$. The same parameters than on Fig. 7 were used.

## 3 References

Akerboom, T. P., Bilzer, M., and Sies, H. (1982). The relationship of biliary glutathione disulfide efflux and intracellular glutathione disulfide content in perfused rat liver. *Journal of Biological Chemistry* 257, 4248–4252. doi: 10.1016/S0021-9258(18)34713-6.

Allali-Hassani, A., Pan, P. W., Dombrovski, L., Najmanovich, R., Tempel, W., Dong, A., et al. (2007). Structural and Chemical Profiling of the Human Cytosolic Sulfotransferases. *PLoS Biol* 5, e97. doi: 10.1371/journal.pbio.0050097.

Armstrong, R. N. (1991). Glutathione S-transferases: reaction mechanism, structure, and function. *Chem. Res. Toxicol.* 4, 131–140. doi: 10.1021/tx00020a001.

Banerjee, R. K., and Roy, A. B. (1968). Kinetic studies of the phenol sulphotransferase reaction. *Biochimica et Biophysica Acta (BBA) - Enzymology* 151, 573–586. doi: 10.1016/0005-2744(68)90004-1.

Bock, K. W., and White, I. N. H. (1974). UDP-glucuronyltransferase in Perfused Rat Liver and in Microsomes: Influence of Phenobarbital and 3-Methylcholanthrene. *Eur J Biochem* 46, 451–459. doi: 10.1111/j.1432-1033.1974.tb03638.x.

Boissier, N., Drasdo, D., and Vignon-Clementel, I. E. (2021). Simulation of a detoxifying organ function: Focus on hemodynamics modeling and convection-reaction numerical simulation in microcirculatory networks. *Int J Numer Meth Biomed Engng* 37. doi: 10.1002/cnm.3422.

Cellière, G. (2016). Multi-scale modeling of hepatic drug toxicity and its consequences on ammonia detoxification.

Chapman, E., Best, M. D., Hanson, S. R., and Wong, C.-H. (2004). Sulfotransferases: Structure, Mechanism, Biological Activity, Inhibition, and Synthetic Utility. *Angew. Chem. Int. Ed.* 43, 3526–3548. doi: 10.1002/anie.200300631.

Coles, B., Wilson, I., Wardman, P., Hinson, J. A., Nelson, S. D., and Ketterer, B. (1988). The spontaneous and enzymatic reaction of N-acetyl-p-benzoquinonimine with glutathione: a stopped-flow kinetic study. *Arch Biochem Biophys* 264, 253–260. doi: 10.1016/0003-9861(88)90592-9.

Conway, J. G., Kauffman, F. C., and Thurman, R. G. (1985). Effect of glucose on 7-hydroxycoumarin glucuronide production in periportal and pericentral regions of the liver lobule. *Biochem J* 226, 749–756. doi: 10.1042/bj2260749.

Dai, G., He, L., Chou, N., and Wan, Y.-J. Y. (2006). Acetaminophen Metabolism Does Not Contribute to Gender Difference in Its Hepatotoxicity in Mouse. *Toxicological Sciences* 92, 33–41. doi: 10.1093/toxsci/kfj192.

Dalhoff, K., and Poulsen, H. E. (1992). Effects of cysteine and acetaminophen on the syntheses of glutathione and adenosine 3’-phosphate 5’-phosphosulfate in isolated rat hepatocytes. *Biochem Pharmacol* 44, 447–454. doi: 10.1016/0006-2952(92)90435-l.

Davies, B., and Morris, T. (1993). Physiological Parameters in Laboratory Animals and Humans. *Pharmaceutical Research* 10.

Drew, B., and Leeuwenburgh, C. (2003). Method for measuring ATP production in isolated mitochondria: ATP production in brain and liver mitochondria of Fischer-344 rats with age and caloric restriction. *American Journal of Physiology-Regulatory, Integrative and Comparative Physiology* 285, R1259–R1267. doi: 10.1152/ajpregu.00264.2003.

Duffel, M. W., and Jakoby, W. B. (1981). On the mechanism of aryl sulfotransferase. *Journal of Biological Chemistry* 256, 11123–11127. doi: 10.1016/S0021-9258(19)68565-0.

Ghallab, A., Cellière, G., Henkel, S. G., Driesch, D., Hoehme, S., Hofmann, U., et al. (2016). Model-guided identification of a therapeutic strategy to reduce hyperammonemia in liver diseases. *Journal of Hepatology* 64, 860–871. doi: 10.1016/j.jhep.2015.11.018.

Guibert, R., Fonta, C., and Plouraboué, F. (2010). Cerebral Blood Flow Modeling in Primate Cortex. *J Cereb Blood Flow Metab* 30, 1860–1873. doi: 10.1038/jcbfm.2010.105.

Hammad, S., Hoehme, S., Friebel, A., von Recklinghausen, I., Othman, A., Begher-Tibbe, B., et al. (2014). Protocols for staining of bile canalicular and sinusoidal networks of human, mouse and pig livers, three-dimensional reconstruction and quantification of tissue microarchitecture by image processing and analysis. *Arch Toxicol* 88, 1161–1183. doi: 10.1007/s00204-014-1243-5.

Hansen, N. (2006). "The CMA Evolution Strategy: A Comparing Review," in *Towards a New Evolutionary Computation: Advances in the Estimation of Distribution Algorithms* Studies in Fuzziness and Soft Computing., eds. J. A. Lozano, P. Larrañaga, I. Inza, and E. Bengoetxea (Berlin, Heidelberg: Springer), 75–102. doi: 10.1007/3-540-32494-1_4.

Hansen, N., and Ostermeier, A. (1996). Adapting arbitrary normal mutation distributions in evolution strategies: the covariance matrix adaptation. in *Proceedings of IEEE International Conference on Evolutionary Computation* (Nagoya, Japan: IEEE), 312–317. doi: 10.1109/ICEC.1996.542381.

Hjelle, J. J., Hazelton, G. A., and Klaassen, C. D. (1985). Acetaminophen decreases adenosine 3'-phosphate 5'-phosphosulfate and uridine diphosphoglucuronic acid in rat liver. *Drug Metab Dispos* 13, 35–41.

Hochman, Y., Zakim, D., and Vessey, D. A. (1981). A kinetic mechanism for modulation of the activity of microsomal UDP-glucuronyltransferase by phospholipids. Effects of lysophosphatidylcholines. *Journal of Biological Chemistry* 256, 4783–4788. doi: 10.1016/S0021-9258(19)69321-X.

Hoehme, S., Brulport, M., Bauer, A., Bedawy, E., Schormann, W., Hermes, M., et al. (2010). Prediction and validation of cell alignment along microvessels as order principle to restore tissue architecture in liver regeneration. *Proc. Natl. Acad. Sci. U.S.A.* 107, 10371–10376. doi: 10.1073/pnas.0909374107.

Howell, B. A., Yang, Y., Kumar, R., Woodhead, J. L., Harrill, A. H., Clewell, H. J., et al. (2012). In vitro to in vivo extrapolation and species response comparisons for drug-induced liver injury (DILI) using DILIsym$^{TM}$: a mechanistic, mathematical model of DILI. *J Pharmacokinet Pharmacodyn* 39, 527–541. doi: 10.1007/s10928-012-9266-0.

Hu, J. J., Lee, M. J., Vapiwala, M., Reuhl, K., Thomas, P. E., and Yang, C. S. (1993). Sex-related differences in mouse renal metabolism and toxicity of acetaminophen. *Toxicol Appl Pharmacol* 122, 16–26. doi: 10.1006/taap.1993.1167.

Huang, Z.-Z., Li, H., Cai, J., Kuhlenkamp, J., Kaplowitz, N., and Lu, S. C. (1998). Changes in glutathione homeostasis during liver regeneration in the rat. *Hepatology* 27, 147–153. doi: 10.1002/hep.510270123.

Iida, S., Mizuma, T., Sakuma, N., Hayashi, M., and Awazu, S. (1989). Transport of acetaminophen conjugates in isolated rat hepatocytes. *Drug Metab Dispos* 17, 341–344.

Irvine, J. D., Takahashi, L., Lockhart, K., Cheong, J., Tolan, J. W., Selick, H. E., et al. (1999). MDCK (Madin-Darby Canine Kidney) Cells: A Tool for Membrane Permeability Screening. *Journal of Pharmaceutical Sciences* 88, 28–33. doi: 10.1021/js9803205.

Jaeschke, H., McGill, M. R., and Ramachandran, A. (2012). Oxidant stress, mitochondria, and cell death mechanisms in drug-induced liver injury: Lessons learned from acetaminophen hepatotoxicity. *Drug Metabolism Reviews* 44, 88–106. doi: 10.3109/03602532.2011.602688.

Kim, H. J., Madhu, C., Cho, J. H., and Klaassen, C. D. (1995). In vivo modification of 3'-phosphoadenosine 5'-phosphosulfate and sulfate by infusion of sodium sulfate, cysteine, and methionine. *Drug Metab Dispos* 23, 840–845.

Klaassen, C. D., Casarett, L. J., and Doull, J. (2013). *Casarett and Doull's toxicology: the basic science of poisons*. 8th ed. New York: McGraw-Hill Professional.

Kraus, P. (1980). Resolution, Purification and Some Properties of Three Glutathione Transferases from Rat Liver Mitochondria. *Hoppe-Seyler´s Zeitschrift für physiologische Chemie* 361, 9–16. doi: 10.1515/bchm2.1980.361.1.9.

Leskovac, V. (2004). *Comprehensive Enzyme Kinetics*. Boston: Kluwer Academic Publishers doi: 10.1007/b100340.

Liu, L., and Klaassen, C. D. (1996). Different Mechanism of Saturation of Acetaminophen Sulfate Conjugation in Mice and Rats. *Toxicology and Applied Pharmacology* 139, 128–134. doi: 10.1006/taap.1996.0151.

Malfatti, M. A., Kuhn, E. A., Murugesh, D. K., Mendez, M. E., Hum, N., Thissen, J. B., et al. (2020). Manipulation of the Gut Microbiome Alters Acetaminophen Biodisposition in Mice. *Sci Rep* 10, 4571. doi: 10.1038/s41598-020-60982-8.

Miller, M. G., and Jollow, D. J. (1987). Relationship between sulfotransferase activity and susceptibility to acetaminophen-induced liver necrosis in the hamster. *Drug Metab Dispos* 15, 143–150.

Miners, J. O., Lillywhite, K. J., Yoovathaworn, K., Pongmarutai, M., and Birkett, D. J. (1990). Characterization of paracetamol UDP-glucuronosyltransferase activity in human liver microsomes. *Biochem Pharmacol* 40, 595–600. doi: 10.1016/0006-2952(90)90561-x.

Mizuma, T., Hayashi, M., and Awazu, S. (1985). Factors influencing drug sulfate and glucoronic acid conjugation rates in isolated rat hepatocytes: significance of preincubation time. *Biochemical Pharmacology* 34, 2573–2575. doi: 10.1016/0006-2952(85)90548-9.

MPD: Data set: Reed2 Available at: https://phenome.jax.org/projects/Reed2.

Nagar, S., Walther, S., and Blanchard, R. L. (2006). Sulfotransferase (SULT) 1A1 Polymorphic Variants $^{*}$1, $^{*}$2, and $^{*}$3 Are Associated with Altered Enzymatic Activity, Cellular

Phenotype, and Protein Degradation. *Mol Pharmacol* 69, 2084–2092. doi: 10.1124/mol.105.019240.

Patten, C. J., Thomas, P. E., Guy, R. L., Lee, M., Gonzalez, F. J., Guengerich, F. P., et al. (1993). Cytochrome P450 enzymes involved in acetaminophen activation by rat and human liver microsomes and their kinetics. *Chem. Res. Toxicol.* 6, 511–518. doi: 10.1021/tx00034a019.

Pezzola, S., Antonini, G., Geroni, C., Beria, I., Colombo, M., Broggini, M., et al. (2010). Role of Glutathione Transferases in the Mechanism of Brostallicin Activation. *Biochemistry* 49, 226–235. doi: 10.1021/bi901689s.

Phillips, M. F., and Mantle, T. J. (1991). The initial-rate kinetics of mouse glutathione S-transferase YfYf. 275, 7.

Prescott, L. F., and Wright, N. (1973). The effects of hepatic and renal damage on paracetamol metabolism and excretion following overdosage.: A pharmacokinetic study. *British Journal of Pharmacology* 49, 602–613. doi: 10.1111/j.1476-5381.1973.tb08536.x.

Reinke, L. A., Belinsky, S. A., Evans, R. K., Kauffman, F. C., and Thurman, R. G. (1981). Conjugation of p-nitrophenol in the perfused rat liver: the effect of substrate concentration and carbohydrate reserves. *J Pharmacol Exp Ther* 217, 863–870.

Riches, Z., Bloomer, J., Patel, A., Nolan, A., and Coughtrie, M. (2009). Assessment of cryopreserved human hepatocytes as a model system to investigate sulfation and glucuronidation and to evaluate inhibitors of drug conjugation. *Xenobiotica* 39, 374–381. doi: 10.1080/00498250902763440.

Schliess, F., Hoehme, S., Henkel, S. G., Ghallab, A., Driesch, D., Böttger, J., et al. (2014). Integrated metabolic spatial-temporal model for the prediction of ammonia detoxification during liver damage and regeneration. *Hepatology* 60, 2040–2051. doi: 10.1002/hep.27136.

Secomb, T. W. (2017). Blood Flow in the Microcirculation. *Annual Review of Fluid Mechanics* 49, 443–461. doi: 10.1146/annurev-fluid-010816-060302.

Secomb, T. W., and Pries, A. R. (2013). Blood viscosity in microvessels: Experiment and theory. *Comptes Rendus Physique* 14, 470–478. doi: 10.1016/j.crhy.2013.04.002.

Sekura, R. D., and Jakoby, W. B. (1979). Phenol sulfotransferases. *Journal of Biological Chemistry* 254, 5658–5663. doi: 10.1016/S0021-9258(18)50465-8.

Sigal, A., Milo, R., Cohen, A., Geva-Zatorsky, N., Klein, Y., Liron, Y., et al. (2006). Variability and memory of protein levels in human cells. *Nature* 444, 643–646. doi: 10.1038/nature05316.

Singh, J., and Schwarz, L. R. (1981). Dependence of glucuronidation rate on UDP-glucuronic acid levels in isolated hepatocytes. *Biochemical Pharmacology* 30, 3252–3254. doi: 10.1016/0006-2952(81)90528-1.

Sohlenius-Sternbeck, A.-K. (2006). Determination of the hepatocellularity number for human, dog, rabbit, rat and mouse livers from protein concentration measurements. *Toxicology in Vitro* 20, 1582–1586. doi: 10.1016/j.tiv.2006.06.003.

Spencer, S. L., Gaudet, S., Albeck, J. G., Burke, J. M., and Sorger, P. K. (2009). Non-genetic origins of cell-to-cell variability in TRAIL-induced apoptosis. *Nature* 459, 428–432. doi: 10.1038/nature08012.

Studenberg, S. D., and Brouwer, K. L. (1993). Effect of phenobarbital and p-hydroxyphenobarbital glucuronide on acetaminophen metabolites in isolated rat hepatocytes: use of a kinetic model to examine the rates of formation and egress. *J Pharmacokinet Biopharm* 21, 175–194. doi: 10.1007/BF01059769.

Sweeny, D. J., and Reinke, L. A. (1988). Sulfation of acetaminophen in isolated rat hepatocytes. Relationship to sulfate ion concentrations and intracellular levels of 3’-phosphoadenosine-5’-phosphosulfate. *Drug Metab Dispos* 16, 712–715.

Tyapochkin, E., Cook, P. F., and Chen, G. (2008). Isotope Exchange at Equilibrium Indicates a Steady State Ordered Kinetic Mechanism for Human Sulfotransferase. *Biochemistry* 47, 11894–11899. doi: 10.1021/bi801211t.

Xie, C., Wei, W., Zhang, T., Dirsch, O., and Dahmen, U. (2014). Monitoring of Systemic and Hepatic Hemodynamic Parameters in Mice. *J Vis Exp*, 51955. doi: 10.3791/51955.

Zhivkov, V., Tosheva, R., and Zhivkova, Y. (1975). Concentration of uridine diphosphate sugars in various tissues of vertebrates. *Comparative Biochemistry and Physiology Part B: Comparative Biochemistry* 51, 421–424. doi: 10.1016/0305-0491(75)90032-2.
